# Time-Domain Investigations of Coherent Phonons in van der Waals Thin Films

**DOI:** 10.3390/nano10122543

**Published:** 2020-12-17

**Authors:** Fabien Vialla, Natalia Del Fatti

**Affiliations:** Institut Lumière Matière UMR 5306, Université Claude Bernard Lyon 1, CNRS, Université de Lyon, F-69622 Villeurbanne, France; natalia.del-fatti@univ-lyon1.fr

**Keywords:** acoustics, coherent phonon, breathing mode, picosecond ultrasonics, time-domain spectroscopy, pump-probe, van der Waals, two-dimensional materials, layered materials, mechanical resonator

## Abstract

Coherent phonons can be launched in materials upon localized pulsed optical excitation, and be subsequently followed in time-domain, with a sub-picosecond resolution, using a time-delayed pulsed probe. This technique yields characterization of mechanical, optical, and electronic properties at the nanoscale, and is taken advantage of for investigations in material science, physics, chemistry, and biology. Here we review the use of this experimental method applied to the emerging field of homo- and heterostructures of van der Waals materials. Their unique structure corresponding to non-covalently stacked atomically thin layers allows for the study of original structural configurations, down to one-atom-thin films free of interface defect. The generation and relaxation of coherent optical phonons, as well as propagative and resonant breathing acoustic phonons, are comprehensively discussed. This approach opens new avenues for the in situ characterization of these novel materials, the observation and modulation of exotic phenomena, and advances in the field of acoustics microscopy.

## 1. Introduction

**Figure 1:**
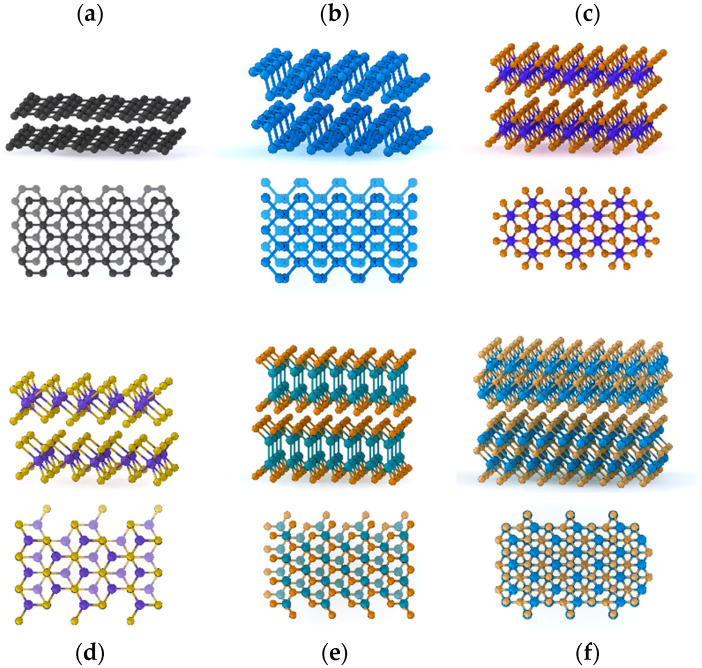
Schematics of a relevant subset of van der Waals materials, which will be discussed in the following review. (**a**) Graphene layers in a Bernal stacking. (**b**) Black phosphorous. (**c**) PtSe_2_ in a 1T phase stacking. (**d**) MoS_2_ in a 2H phase stacking. (**e**) InSe in a γ phase stacking. (**f**) Bi_2_Te_3_.

Van der Waals (vdW) materials, also referred to as layered two-dimensional (2D) materials, are strongly anisotropic materials formed by layers of covalently bound atoms, stacked on top of each other and linked through vdW forces. Many lattice structures are encountered for the 2D layers, as depicted in Figure 1, such as hexagonal one-atom-thick layers in graphite (where monolayers are referred to as graphene) and boron nitride (hBN), two-atom-thick layers in black phosphorous (BP), three-atom-thick layers with octahedral (PbI_2_, PtSe_2_…) or trigonal prismatic coordination (MoS_2_, WSe_2_…) usually referred to as transition metal dichalcogenides (TMDs), four-atom-thick layers (GaS, InSe…), five-atom-thick layers (Sb_2_Se_3_, Bi_2_Te_3_…) usually referred to as V_2_VI_3_ chalcogenides or as quintuple layers (QLs), and many more complex structures [1,2]. In addition, they present various phases, also referred to as polytypes, related to the stacking alignment order of the layers, which further influence their physical properties [2,3]. In sum, the vdW material family spans the full variety of material classes, from dielectrics to metals, semiconductors with bandgaps from ultraviolet to mid-infrared, ferromagnets, piezoelectrics, superconductors, topological insulators, etc. [4,5,6,7,8]. Importantly, their strong structural anisotropy affects their mechanical, electronic, optical, and thermal properties. These original bulk properties have been under intense characterization during the 1970s and 1980s [1,2,9].

A fresh appeal sprouted out in 2004 with the exploration of vdW materials reduced to a single monolayer after the first mechanical exfoliation and characterization of graphene from bulk graphite [10,11]. Its unconventional structure, a honeycomb lattice of carbon atoms forming a true 2D layer with no dandling bonds out of the plane, is at the origin of many unique features. The most striking one may be the gapless, massless Dirac electrons [10] from which arise a spectrally flat optical absorption [12] and many exotic quantum transport regimes [13]. Graphene also presents superior thermal [14] and mechanical properties [15]. Likewise, the whole vdW materials family can be thinned down to a single layer, yet sometimes at the expense of reduced chemical stability. When reduced to the atomic scale on the out-of-plane dimension, most of these materials present emerging original properties stemming from quantum confinement and weakly screened charge carriers, for instance room-temperature excitonic behaviors in semiconducting TMDs [16,17]. Additionally, since single-layer materials are only composed of surface atoms, they present a deep tunability of their properties through their environment, for instance via capacitive coupling [10,18] and Coulomb screening [19]. However, the coupling to an uncontrolled environment, e.g., to a substrate or adsorbed molecules, usually predominates over the intrinsic properties. Thus, extrinsically induced defects, inhomogeneities, and a dominant energy loss to the substrate were very detrimental in early studies [20,21,22].

It is to circumvent these issues that the concept of vdW heterostructures was introduced. Graphene monolayer was first transferred on top of hBN [23], and then encapsulated in-between [24], to achieve passivation and flattening of the layer. This principle of stacking different layers on top of each other has been extended over the years to the whole family of vdW materials [6,8]. Major nanofabrication challenges have been overcome, in particular concerning the cleanliness of the interfaces to achieve large scale, homogenous, optimized coupling [25,26,27]. Selective electrical contacting [28] and control over the relative angle between the layer lattices [29] can be implemented. In parallel, huge improvements have been obtained in the field of material synthesis leading to the successful growth of large-scale, single-crystal layers [30,31]. Chemical exfoliation and sorting processes yield large-scale, high-quality, controllable ensembles of few-layer sheets [32,33]. Moreover, vdW heterostructures can be directly grown via chemical vapor deposition or molecular beam epitaxy [8,34], rather than tedious exfoliation and subsequent stacking of the layers. VdW heterostructure versatility can be further expanded by intercalating atoms or molecules between the layers, or functionalizing the layer surfaces [35,36]. In particular, changing growth stoichiometry of QL materials yields natural superlattices with for instance intercalation of Bi_2_ vdW layers in Bi_2_Se_3_ stacks, or alternation of Bi_2_Te_3_ and Sb_2_Te_3_ layers with controllable periodicity [37,38,39]. Overall, this brings interesting potentials for scalable manufacturing and integration with the current CMOS technology [40]. In sum, state of the art nanofabrication now yields heterostructures, i.e., composite vdW materials with atomic precision on the out-of-plane dimension, which can be processed as scalable and in situ tunable devices. This corresponds to a unique platform in fundamental material science to study the basic features of a vdW interface and engineer emerging exotic effects.

In this review, we provide a contextualized panorama of the rising field of coherent phonon investigation in vdW systems. In Section 2, we comprehensively introduce the fundamentals on the structural and mechanical properties of vdW homo- and heterostructures. Particular attention will be given to the quantitative characterization of the interlayer vdW coupling. In Section 3, we present a general overview on the ultrafast physics at play in the launching, propagation, and relaxation of coherent phonons in time-domain experiments. Relevant examples in various systems will be depicted to provide the full scope of possibilities opened by this wide and mature field of research. In Section 4, we thoroughly review and discuss the experimental studies reported in the recent years on time-domain investigation of coherent phonons in thick and thin slabs of vdW materials. The variety of observed acoustic waves in the GHz and THz regimes is comprehensively described in terms of acoustic or optical modes, which can be bulk-like, propagative, or resonant when confined to the nanoscale. Quantitative analysis and comparison of extracted frequencies and quality factors enable the study of the vdW mechanical coupling at homo- and hetero-interfaces. Future perspectives are finally discussed building on the possible overcoming of current experimental limitations and on still-unexplored routes.

## 2. Mechanical Properties of van der Waals Materials

Given the large variety of existing vdW materials, a thorough review of each specific structural configuration is far beyond the scope of this section. In the following, we propose a general overview of the features related to the structural properties, phonon modes, and mechanical behaviors that are shared by all vdW materials. We present fundamental knowledge using the experimental probing techniques as a guideline. The reader interested in more exhaustive descriptions of the different vdW material families and their specific properties can refer to general reviews [4,5,6,7,8] and the more specific ones referenced throughout the following discussion.

### 2.1. Raman-Active Phonons

Here we primarily focus on Raman spectroscopy, given its wide use in the characterization of vdW materials. Raman spectroscopy is an all-optical experimental technique that relies on the inelastic scattering of light in a probed material. Specific selection rules in this process yield the involvement of so-called Raman-active phonons with specific symmetries, which mostly correspond to a subset of optical phonons at given points of the Brillouin zone. The technique corresponds to a fast, non-invasive probe with a sub-micron spatial resolution, which provides structural and electronic information, and is particularly well suited for small area 2D systems such as thinned down vdW materials. Several reviews explore the richness of this experimental approach [41,42,43,44,45,46], which will be readily overviewed here.

As all fundamental properties of vdW materials, phonon properties are naturally ruled by the large structural anisotropy stemming from the contrast between the strong covalent intralayer bonds and the soft vdW out-of-plane coupling between layers. Therefore, phonons can easily be discriminated between *intralayer* and *interlayer* modes from their harder (i.e., higher) and softer (i.e., lower) frequencies, respectively [2]. Within the vdW materials family, the layers of covalently bond atoms can take a large variety of structures, from one to several-atom-thick layers and even more complicated clustered structures. Therefore, intralayer modes span a very large set of frequencies and symmetries. Nevertheless, they all appear in the THz regime. Strong bonds and light atoms, as it is the case in graphene [47,48] and hBN [49,50], yield the hardest optical modes in Raman around 50 THz (around 1500 cm^−1^), while softer modes, usually related to heavier atoms like in TMDs [51,52], can go down to around 10 THz (around 300 cm^−1^). Due to their intralayer nature, they only present a slight dependence with the number of layers and deviate from the bulk value only close to the monolayer case.

**Figure 2:**
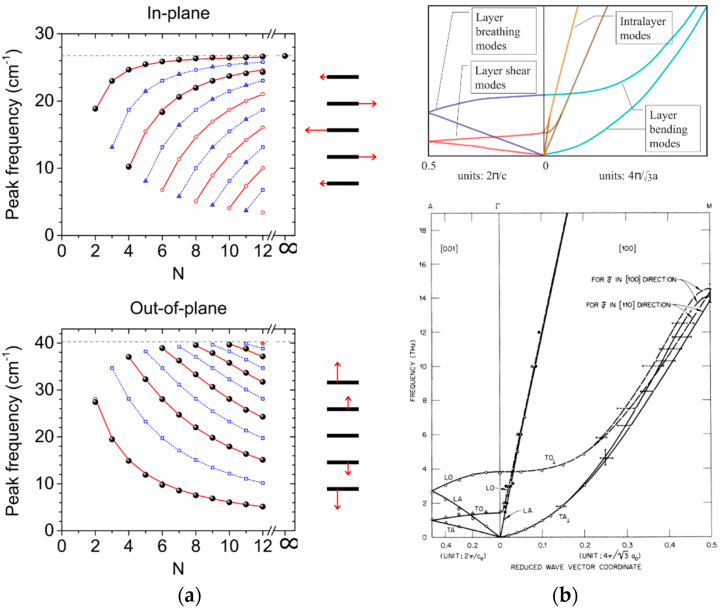
(**a**) Energy of the Raman-active interlayer phonon modes, (top) shear and (bottom) breathing, as function of the number of layers in MoTe_2_. Schematics of the displacement of each prominent mode is shown in the five-layer case. Adapted from [52], with permission from American Chemical Society, Copyright ©2015. (**b**) (top) Phonon dispersion schematic for layered materials. From [62] © IOP Publishing. Reproduced with permission. All rights reserved. (bottom) Experimental phonon dispersion from inelastic-neutron-scattering spectroscopy in graphite. Reprinted with permission from [63], Copyright ©1972 by the American Physical Society.

In contrast, interlayer phonons appear at lower frequencies, in the hundreds of GHz range up to around 1 THz, due to the weak vdW coupling between layers. They are thus quite specific to vdW materials and will be of high interest when the case of coherent phonons is discussed. They can be separated in two groups corresponding to different displacement directions, namely the out-of-plane *breathing* modes [53,54] and the in-plane *shear* modes [55,56]. Interestingly, these modes can be accurately described using simple atomistic models of masses and springs [52,54]. This allows to intuitively analyze them in terms of classical coupled oscillators when the number of layers is reduced, yielding quantized modes whose frequencies deviate from the one in bulk, as shown in Figure 2a. We note that among the breathing modes, the ones with lowest index, and therefore lowest frequency, are preferentially observed. The corresponding fundamental dominant breathing mode consists in the out-of-phase oscillation of two blocks composed of half of the layers in the crystal. In contrast, shear modes with highest index and highest frequency prevail. The dominant shear mode consists in the out-of-phase displacement of adjacent layers. This explains their main contrasting Raman features, for instances the respective softening and hardening of observed breathing and shear modes when number of layers are increased, and the silent nature in bulk of the former in contrary to the latter [2,44,52,54]. Finally, we note that the frequency of the Raman-active breathing modes is significantly more sensitive to the number of layers than for the one of shear modes. They are hence preferentially used for crystal thickness evaluation.

Overall, signals from both intralayer and interlayer modes in Raman spectroscopy are widely used in vdW materials for sample characterization, primarily of the structural properties [44,45,46]. This includes many properties of the layers such as lattice orientation, anisotropy, strain, stoichiometry, disorder, type and quality of edges, etc. The stacking order can also be probed in terms of relative lattice translation [57] and rotation [29] between layers. Moreover, since Raman spectroscopy relies on a scattering of light by matter, it also probes electronic properties, such as the band structure, doping level, and electron-phonon coupling [42,58]. Although all the previously discussed features concern few-layer and bulk forms of single vdW materials, here we wish to remark that similar interlayer coupling also emerges in heterostructures and have been probed with Raman spectroscopy. Hybrid breathing and shear modes have been observed in different type of hetero-bilayers of TMDs [59]. Shift and broadening of Raman modes of graphene lying on top of a hBN layer have brought evidence for lattice stretching of the former to adapt to the slightly larger lattice periodicity of the latter, constituting a so-called commensurate state [60]. Phonons in hBN have been probed using an electronic transition resonance from WSe_2_ when the two layers are stacked on top of each other [61].

### 2.2. Phonon Dispersion

The thorough study of phonon dispersion in thin vdW materials is not straightforward mainly due to the limitations in the experimental techniques available. Firstly, Raman spectroscopy almost exclusively probes optical phonons due to its selection rules. However, we note that a double resonant Raman scheme allows to indirectly reconstruct part of the in-plane acoustic phonon branches [64]. Secondly, the optical spectroscopy that relies on the inelastic scattering by acoustic phonons is Brillouin spectroscopy. Applied to bulk vdW materials, the signal appears dominated by surface Rayleigh waves [65,66]. Bulk-like acoustic phonon modes can only be observed when the crystal is transparent enough at the probe wavelength [65] or reduced in thickness to a few layers [67]. However, only in-plane longitudinal and transverse mode branches are extracted. To gain access to all branches, and especially the ones with propagation and/or displacement in the out-of-plane direction, other experimental techniques involving the inelastic scattering of neutrons [9,63,68,69], electrons [70,71], or X-rays [72,73] need to be utilized. We note that these techniques are significantly more demanding than optical spectroscopies in terms of experimental setup and sample size, and may not be compatible with the in situ probing of crystals reduced in thickness and area.

Overall, typical bulk phonon dispersion, shown in Figure 2b, is deeply molded by the strong anisotropy of vdW materials [62]. Acoustic modes with propagation and displacement in the in-plane direction consistently present a much larger velocity than modes in the out-of-plane direction. Modes propagating in this latter direction, with a displacement either longitudinal or transverse, can be discriminated between breathing and shear modes, respectively, in accordance with the previously introduced denomination. We also bring focus to the specific case of acoustic modes propagating in the plane with a transverse out-of-plane displacement. Due to the weak vdW interlayer coupling, their effective velocity is strongly reduced, and their dispersion branch can be well approximated with a quadratic dispersion [62]. They are referred to as bending or ripple modes in the literature, depending on whether long or short acoustic wavelengths are considered, respectively.

Quantitatively, the case of graphite is well known for its very high in-plane sound velocity, with longitudinal and transverse values in the range of 21 and 14 km/s, respectively, which slightly vary depending on the stacking arrangement and number of layers [63,64,67,73]. In strong contrast, breathing and shear modes present velocities around one order of magnitude lower in graphite. The anisotropy is less pronounced in other materials such as TMDs [9,65,68,69], usually because of slower in-plane modes due to heavier atoms.

The full dispersion of the bulk acoustic and optical phonon branches can be reconstructed from the previously discussed structural measurements and compared to theory. Calculations from first principles usually reproduce experimental data well for vdW materials and enable the identification of the different phonon branches, as presented in Figure 2b [9,63,74]. Standard approaches have been utilized for the evaluation of the dynamical matrix specific to each crystal structure, by explicitly introducing force constant parameters (valence force field model and its approximations for given symmetries) and fitting to experimental data in early works [9,63], and recently with more complex computations for instance in the density functional theory (DFT) [74,75,76]. Away from the zone center, deviations from simple first principle calculations can appear in measurements. Effects of electron-phonon and electron-electron coupling, enhanced by the weak Coulomb screening in a layered structure, must be taken into account [77,78]. For instance, Kohn anomalies are observed in different metallic vdW materials [69,77].

A thorough discussion on the electron-phonon and phonon-phonon couplings in vdW materials is beyond the scale of this review. Yet, we again emphasize that their features naturally stem from the structural anisotropy and the reduced dimensionality when the crystal is thinned down. Electron-phonon and phonon-phonon couplings are of key importance in many physical mechanisms, such as electronic transport [13,23,79,80] and optical [81] processes where they offer specific inelastic and elastic scattering paths. They also drive the thermal properties, which can be strongly influenced by phonon confinement effects [82,83] and the nature of the nanoscale bonds [84]. Specific reviews on vdW materials offer exhaustive description of the physical thermal features, their theoretical evaluation, and experimental probing [82,85,86]. Structural anisotropy plays again a major role. Out-of-plane thermal conductivities appear lower by usually more than one order of magnitude compared to in-plane ones in vdW crystals [87,88]. Overall, we note that processes involving acoustic and optical phonons are still under intense fundamental and device-oriented study, in particular when original hybrid interfaces in vdW heterostructures are addressed.

### 2.3. Static and Dynamical Stress

The mechanical properties of vdW systems can be efficiently probed by investigating their response under a spatially and temporally coherent external stress. Such studies are presented in detail in recent reviews [89,90,91]. In particular, the possibility for vdW materials to be thinned down to a monolayer without introducing structural defects has motivated their study as ultrathin flexible membranes. Graphene, which combines strong covalent bonds and light atoms, presents superior mechanical properties and has attracted much interest. A brief review of general mechanical properties of vdW materials in bulk and few-layers forms is presented in the following, with special focus on the effects of the interlayer vdW coupling.

Key mechanical parameters are the elastic moduli, which correspond to the linear coefficients between uniaxial external stress and internal strain in a material. For monolayers, the 2D in-plane elastic modulus has been quantitatively evaluated from many different experimental schemes [89,90,91]. The most direct one corresponds to the local indentation with an atomic force microscope tip of a suspended membrane. This *static* stress yields bond stretching and negligible bending. Values for the 2D in-plane elastic modulus of 330 N/m [15], 300 N/m [92], and around 180 N/m [93,94] are extracted for graphene, hBN, and TMD monolayers, respectively. This 2D modulus is compared to the bulk in-plane Young’s modulus (which corresponds to the $C_{11}$ component of the bulk elastic moduli matrix) by assuming the thickness of a monolayer equal to the interlayer distance in the bulk structure. For monolayer graphene, the bulk modulus of graphite $C_{11}$ = 1 TPa is retrieved, which evidences the high quality of available crystal of highly oriented pyrolytic graphite. In contrast, other bulk vdW materials usually present slightly lower Young’s modulus compared to their monolayer case [91,94], which is interpreted as a sign of stacking faults. However, we note that these observations are strongly sample dependent [92]. Interestingly, indentation study on bilayers of TMDs shows similar behaviors in artificial heterostructures and natural homolayer stacks, confirming that similar vdW interaction is at play [94].

Investigation of the mechanical response in the out-of-plane direction is experimentally more demanding. For instance, indentation studies require a significantly higher resolution to resolve interlayer bond compression [95]. Out-of-plane Young’s modulus (which corresponds to the $C_{33}$ component of the bulk elastic moduli matrix) of graphite and few-layer graphene has been extracted, with a value around 35 GPa in agreement with structural bulk data. An interesting alternative consists in applying a global high pressure to vdW systems in a diamond anvil cell [96,97,98,99]. The mechanical response is mostly followed using the characterization versatility of Raman spectroscopy, as discussed in Section 2.1. The hardening of the phonon modes for increasing pressure is consistently observed, with a significantly stronger relative effect on the out-of-plane modes. At high pressures in the GPa range, interlayer distances are reduced and the vdW material progressively loses its anisotropic structure [99]. In the case of few layers under high static pressure, the mechanical and chemical environmental effects related to the substrate and the pressure transmitting medium must be considered [100,101].

More generally, Raman spectroscopy is utilized for the non-invasive characterization of structural, electronic, and optical changes induced by external mechanical stress. Many investigations concern thinned down vdW materials deposited on bendable and stretchable substrate, for fundamental studies and towards flexible applications where tunability of physical features is sought [102,103,104,105]. We note that, from these schemes, strong piezoelectricity has been experimentally observed for monolayer and odd number of layers of specific vdW materials [106].

Alternatively, *dynamical* studies offer a fruitful probe of mechanical properties in a regime complementary to previously discussed *static* investigations. We first mention the ultrasonic pulse-echo technique applied to macroscopic bulk vdW materials [107,108,109]. Acoustic transducers, electronically triggered and read out with a repetition rate in the MHz range, are utilized to launch strain waves which propagate inside the probed sample. Measurement of the travel time through the echo signal delay yields the evaluation of the sound velocities, in agreement with structural investigations. However, this experimental scheme is not directly applicable to thin vdW slabs due to their smaller areas and shorter acoustic travel times. In this case, suspension of a vdW thin membrane over a conductive substrate allows the actuation and reading out of its bending motion from capacitive coupling. Mechanical oscillations occur in the 100 MHz regime for typical micron size membranes, and hence can be followed coherently in real time with standard fast electronics [110,111]. Values of in-plane Young’s modulus can be extracted similarly to static indentation studies. Interestingly, a transition of behaviors from membrane-like (where in-plane elasticity and pre-tension dominate) for mono and few layers, to plate-like (where bending rigidity dominates) for thicker crystals with typically more than 10 layers was evidenced [112]. The quality factor of such resonator brings a lot of physical information on the system. Even if they still do not reach the figures of merit of state-of-the-art micro and nanopatterned oscillators, membranes of vdW monolayer show great potential for sensing and quantum motion applications [113,114].

Finally, we briefly mention the case of surface acoustic waves, which also lie in the 100 MHz regime. These modes, which are coherently actuated and probed in piezoelectric materials using interdigital transducers, can be coupled to a vdW monolayer deposited on top of it [115]. Experimental investigations have evidenced the good mechanical interaction, which allows coherent tuning of physical parameters of the monolayer [116].

In this context, a need for the simple, precise probing of the out-of-plane acoustic phonons in few- and monolayer vdW systems is identified. High spatial resolution, in-plane but primarily in the out-of-plane direction where deviations from the bulk form are more likely to emerge, is of great interest. Bringing coherent dynamical studies to higher frequencies in the GHz and THz range would enable such fruitful investigations. Moreover, reaching such high frequencies allows to probe the anharmonicities of acoustic modes and bridge the gap with optical modes. Such studies cannot be implemented with ultrafast electronics and require the use of transient technics in the time-domain with resolution up to femtosecond timescales, which will be introduced in Section 3.

### 2.4. Mechanical Parameters

As introduced in the previous sections, various mechanical parameters can be extracted depending on the experimental technique involved, e.g., the energy of specific phonons modes from Raman spectroscopy, the velocities of acoustic phonons from phonon dispersion and ultrasound travel time experiment, the elastic moduli matrix from static stress investigation, and the chemical bond elastic constants (or equivalent spring stiffness) from atomistic modeling. To rationalize the present review and help further discussions, here we quantitatively connect them together, with special focus on the values related to the vdW interaction.

Following the general definition of the elastic moduli matrix with $C_{\mathrm{ij}}$ components, we can relate them to different acoustic phonon branch velocities, such as $C_{11}={\rho v}_{\mathrm{LA}}^{2}$ with v_LA_ being the in-plane longitudinal mode velocity,

(1) $$C_{33}={\rho v}_{B}^{2}$$

with v_B_ being the breathing (or out-of-plane longitudinal) mode velocity and $C_{44}={\rho v}_{S}^{2}$ with vs. being the shear (or out-of-plane transverse) mode velocity, where ρ is the density of the vdW material [9,63,108]. The other introduced parameters require a structure dependent model, such as an atomistic first principle model, to be put in relation. Here we make use of a rigid-plane model, which corresponds to a crude yet relevant approximation and enables universal description of all vdW materials [44,117,118]. Any layered structure is hence simply defined by the following structural properties: Mass per unit area µ and out-of-plane periodicity of the layers d, which verify ρ=µ/d. The mechanical vdW coupling between the layers is parametrized by an effective interlayer elastic constant per unit area K. As for the simple case of a linear chain of springs and masses, an out-of-plane acoustic wave with vector q and frequency f presents the following dispersion relation:

(2) $$f=\frac{1}{\pi }\sqrt{\frac{K}{2\mu }(1-\cos\mathrm{qd})}=\frac{1}{\pi }\sqrt{\frac{K}{\mu }}\sin\left|\frac{\mathrm{qd}}{2}\right|.$$

The long wavelength (or continuous medium) limit, i.e., when qd≪1, gives $f=\sqrt{\frac{K}{\mu }}\frac{\mathrm{qd}}{2\pi }=\frac{v}{\lambda }$, introducing the acoustic wavelength $\lambda =\frac{2\pi }{q}$ and velocity

(3) $$v=\sqrt{\frac{K}{\mu }}d.$$

This model can be applied to both longitudinal (breathing) and transverse (shear) cases. In the following, we will only consider the former case since it is the one generally probed in the time-domain investigations reviewed in Section 4. As already mentioned, more advanced atomistic models found in the literature, which consider first neighbors or more, or the actual bond tridimensional geometry, yield a large set of interatomic elastic constants. From structural considerations, they can be translated into the characteristic interlayer constant K from simple linear combination [52,119].

Parameters v_B_, K and $C_{33}$, which all stem from the same physical vdW coupling between layers, are reported in Table 1 from various investigations. The out-of-plane longitudinal elastic modulus $C_{33}$ shows a relatively small span for all vdW materials, from 30 to 60 GPa. This constitutes a good characterization of the physical nature of the bond between the layers, when compared to Young’s modulus of other materials. For instance, tridimensional vdW crystals such as C_60_ crystal have similar values in the order of 20 GPa [120]. In comparison, covalent crystalline structures present larger Young’s modulus, between 100 and 200 GPa for usual metals and silicon for instance, up to a value of 1 TPa for in-plane covalent bonds of graphite. Softer materials like polymers or organic solids show values below 10 GPa [121,122]. We note that elastic modulus of amorphous materials like glasses might fall in the same range as the out-of-plane one of vdW materials. Among vdW materials, the one-atom-thick ones (graphene, hBN) present the lowest values of $C_{33}$ around 35 GPa, TMDs all have a value around 50 GPa and BP appears as the one with the largest value around 60 GPa. In contrast, one-atom-thick layered materials (due to their low density) and BP share the highest values of v_B_ around 4 to 5 km/s. Other vdW materials generally present velocities around 2 to 3 km/s. We note that TMDs show a monotonous decrease of their acoustic vdW velocity with increasing density. The extracted interlayer stiffness K present values from 4 to 12 × 10^19^ N/m^3^, stiffest materials being the ones with thinnest layers. These values are also characteristics of a 2D vdW coupling. A similar value is evaluated between a graphene monolayer and a SiO_2_ substrate (9.0 × 10^19^ N/m^3^) from roughness conformation investigation [123]. Another quantitative parameter, not reported here, which characterizes a vdW coupling is the binding energy between the layers, with a relatively universal value around 18 meV/Å^2^ [75]. Finally, we note that the out-of-plane periodicity d values follow the ones of the layer thickness, and the typical vdW spacing between the external layer atoms is around 3.0 Å, a value larger than twice the covalent radius of involved atoms (below 1.2 Å).

## 3. Time-Domain Acoustic Investigations

To resolve ultrafast transient phenomena, time-domain investigation exploits a pump-probe experimental scheme where an optical pump pulse puts the studied system out of its equilibrium, and a following probe pulse measures different physical parameters after a controlled time delay. Detection relies on averaging over many shots at fixed delays given that pump and probe are synchronized, in a stroboscopic-like manner. Practically, pulses often originate from the same optical oscillator, and follow different optical paths with one of them being tunable in length using a stepper motor on a translation line. Given the speed of light, a spatial step of a micron approximately corresponds to a 3-femtosecond delay. Therefore, the relaxation phenomena in the investigated sample can be coherently followed in time with a temporal resolution limited by the pulse duration in the femtosecond range. Alternatively, the asynchronous optical sampling technique (ASOPS) that relies on different oscillators locked together with different repetition rate allows similar studies with high-speed scanning by removing the mechanical delay line [144,145].

The pump pulse usually triggers a cascade of relaxation mechanisms, where the energy is transferred from the photons to the electrons and to the (mostly incoherent) phonons of the probed system and its environment. Time-domain investigations are indeed of key relevance for the fundamental study of electron–electron, electron–phonon, and phonon–phonon couplings as well as of ultrafast optoelectronic devices and heat management. Here we comment on the fundamental mechanisms leading to the generation of coherent phonons after pulsed optical excitation, their subsequent evolution, and the corresponding state-of-art probing schemes.

### 3.1. Generation of Coherent Phonons

Pioneer studies on coherently generated and probed phonon modes in thin films emerged in the 1980s, following the development of ultrafast pulsed laser systems and their use in the probing of condensed matter [146,147]. Although the involved physical mechanisms are discussed in early works [148,149,150,151], we primarily guide the reader towards the recent exhaustive review from Ruello et al. [152]. Here we chose not to explicitly derive the formalism of elastic motion in a crystal, but rather to comprehensively describe the competing fundamental mechanisms at play for the launching of coherent phonons.

After absorption of the pump optical pulse, injected energy lies in the electrons of the investigated system. The carrier distribution is modified from the equilibrium case in the form of the population and depopulation of given bands related to different orbitals with different spatial distributions. Therefore, since the interatomic forces rely on the electrostatic interaction between electrons and cations in the crystal, the equilibrium configuration of the lattice is modified. This modification directly corresponds to the launching of a coherent phonon wave. This mechanism, namely the interaction between electrons and lattice ions yielding phonon excitations, is readily described through the *deformation potential* formalism. After or in parallel of this phenomenon, energy can incoherently relax to the phonons, acoustic, and/or optical modes, which subsequently interact with each other to finally form a thermalized distribution with a temperature higher than the one of equilibrium. From the lattice anharmonicities, crystal lattice modification is induced, and coherent phonons are launched. This mechanism is known as *thermoelasticity* and quantitatively relates to the Grüneisen parameter and the heat capacity of the probed materials.

Competition occurs between these two main mechanisms depending on the crystal properties and excitation parameters. For instance, thermoelasticity generally dominates in metals, due to the strong electron–phonon coupling that yields an ultrafast sub-picosecond heating of the lattice, and thus the launching of high frequency coherent acoustic phonons [153]. We note that, for metals with weaker electron-phonon coupling such as gold, a hot carrier distribution builds up and diffuses in the crystal before relaxation to the phonon bath, thus stretching the effective coherent phonon spatial wave envelope [154]. In contrast, the deformation potential effect dominates in semiconductors due to the usual relatively longer lifetimes of excited electron-hole pairs lying at the bandgap edges [155]. Although generally tensile due to the promotion of electrons into anti-bonding bands, the stress stemming from deformation potential can be compressive in some materials [156]. As in the metal case, diffusion of the photo-excited electron-hole plasma can affect the coherent phonon wave spatial extension [157].

Other noteworthy mechanisms can be observed in more specific conditions. In non-centrosymmetric materials, *piezoelectricity* couples the strain in the crystal to a macroscopic electric field. An internal electric field stemming from a static built-in strain can undergo ultrafast screening by pumped photocarriers, which yields the launching of coherent phonons in such materials. This corresponds to the dominating mechanism in engineered structures such as quantum wells [158] or p-n junctions [159]. Finally, coherent phonons can be launched even when the optical pump pulse is not absorbed, though with a weaker efficiency due to the non-resonant interaction. The corresponding mechanism is usually referred to as impulsive *stimulated Raman (Brillouin) scattering* and shares similar selection rules with its spectroscopic counterpart for the launching of optical (acoustic) phonons [151,160,161].

From this brief review, we find that different mechanisms can lead to emission of coherent phonons upon optical pulsed excitation. Structural, optical, electronic, and thermal parameters of the system determine which one is dominating. Experimentally, control over the excitation properties, wavelength, polarization, fluence, and pulse duration can tune and help uncovering the mechanisms effectively at play [152]. Interestingly, the coherent nature of the investigation enables the analysis of the phase at start of the mechanical oscillations. From this, phonon generation mechanisms are discriminated into two types: *Impulsive* and *displacive* [150,162]. The former corresponds to a relatively instantaneous launching, much shorter than the oscillation period, and yields a sine wave. The latter results from an energy injection to the lattice in a timescale shorter yet comparable to the resulting oscillation period, which thus follows a cosine wave response. We note here that if phonon launching occurs with a timescale larger than its typical period, strong dephasing will completely hinder its coherent observation. This further emphasizes the key roles of the pump pulse duration and focusing, and the dynamics of the phonon generation mechanisms in the temporal and spatial shaping of the generated coherent phonon wave packet.

### 3.2. Excited Modes and Detection Scheme

Generally, the pulsed probe is a delayed optical pulse originating from the same pulse as the pump with yet different features such as wavelength (with the use of non-linear optics), polarization, (lower) fluence, incidence angle, spatial position, etc. It can be recollected after reflection on or transmission through the sample. In either case, the collected signal is modulated by the local changes in the dielectric function of the sample, real and imaginary parts, introduced by the coherent phonons. We note that such modifications can also be generated by the electronic and incoherent phonon evolutions in the crystal upon pump excitation. This inherently complexifies the interpretation of the optical signal but also illustrates the richness of information, which can be extracted from time-domain investigations. Alternatively, non-optical pulsed probe can be introduced, given that it is perfectly synchronized with the pump. Usually, it directly originates from a delayed optical pulse, through photoelectric effect in time-domain electron microscopy for instance. Specificities of such schemes will be discussed for actual cases in Section 4.6. Here we describe the nature and properties of the coherent phonons that can be launched and comment on the corresponding optical signal features extracted in time-domain investigation, in particular the frequency f and quality factor Q, defined as Q=πτf where τ is the exponential damping time constant of the oscillating signal.

**Figure 3:**
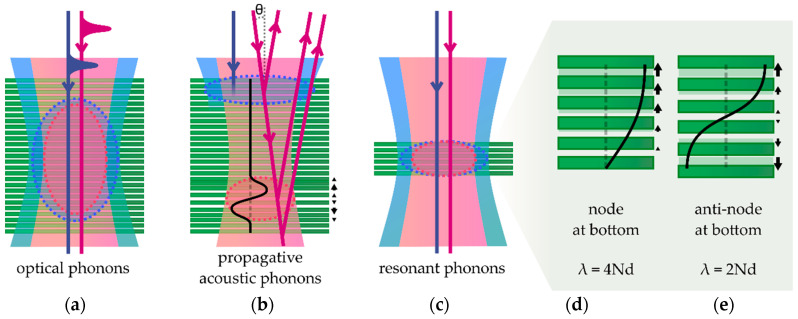
Schematic representations of the time-domain probing of coherent phonons of different nature in a layered material, namely (**a**) optical phonons, (**b**) propagative phonons, and (**c**–**e**) resonant phonons when the film is reduced to the nanoscale. Pump and probe pulsed beams are presented with blue and pink solid lines, respectively, as well as the respective pumped and probed regions with dashed circles in (**a**–**c**). Scattered beams at the top and bottom interfaces, and at the propagative strain wavefront with a resulting different optical paths are shown in (**b**). The spatial acoustic waveform is presented with a solid black line in (**b**,**d**,**e**) along with black arrows indicating the corresponding out-of-plane displacement.

Firstly, coherent optical phonons (COPs) can be generated after either impulsive stimulated Raman scattering or a displacive mechanism following light absorption [150,151,160,161,163]. Since no effective COP propagation occurs, pump and probe must overlap in this configuration, as depicted in Figure 3a. Focusing properties of both beams, namely the lateral extension and penetration depth, define the spatial resolution. Here we have the simplest case where the collected probe beam is directly modulated at the frequency and phase of the COPs in the sample [151,161]. Hence, spectral analysis of the optical signal allows for the identification of the launched mode(s) in the same manner as for standard spectroscopy. Quality factor of the oscillating signal relates to the intrinsic damping of the COPs in the crystal and to the dephasing, which can originate from the spatial and temporal extension of the emission region. Due to the deep similarities with the processes involved in Raman spectroscopy, similar yet complementary information is extracted about optical phonons for the characterization of the investigated system.

In contrast, pulse-generated coherent acoustic phonons (CAPs) can propagate in the system far away from the region where they are launched due to their finite velocity, as presented in Figure 2b [147,164]. Their probing thus provides significantly different information as compared to the study of COPs. In the literature, time-domain investigations involving the study of those propagating CAPs are usually referred to as picosecond ultrasonics or time-domain Brillouin scattering and have found applicative purpose for the high-resolution structural characterization in many fields from material science to biology [148,165,166]. As an example in connection to this review, this technique has already been implemented for the characterization of ordered and disordered supra-structures of nanoparticles linked through vdW bonds [167,168]. To follow the CAP propagation inside the investigated system, the probe pulse is usually taken with a wavelength for which the material is transparent. The optical signal originates from collection of the probe light scattered by the propagating CAPs, which interferes with the probe light reflected by the different immobile interfaces of the sample. The relative phase between these fields varies over time due to their varying relative optical paths, with a linear variation for a constant CAP velocity v. This yields an oscillating sine wave with a constant frequency in the optical signal usually referred to as Brillouin frequency. In the general case of a probe beam with angle θ compared to normal incidence (Figure 3b), one can write

(4) $$f=2v\sqrt{n_{\mathrm{pr}}-\sin^{2}\theta }/\lambda_{\mathrm{pr}}$$

where $\lambda_{\mathrm{pr}}$ and $n_{\mathrm{pr}}$ are the probe wavelength and the optical index of the material at the probe wavelength, respectively. We emphasize that in this regime the extracted frequency in time-domain is not the one of the CAPs but depends on both probing and material parameters.

Advance schemes have been developed over the years. For instance, following Equation (4), tunability of the incident beams’ angle enables the independent extraction of the previously introduced parameters, $n_{\mathrm{pr}}$ and v, and the sample thickness [166,169,170]. Similarly, transverse shear acoustic modes, in place of the usually involved longitudinal compressive (breathing) modes, can be generated and probed using oblique pump and/or probe beams [171,172]. Variations in the amplitude and phase of the oscillating optical signal, rather than variations in the frequency, are usually monitored to extract information on the spatial inhomogeneities of the sample. Slight modifications in the elastic properties can be probed at interfaces between different lattice structures, or in inhomogeneous nanoporous and defective materials, for instance [173,174]. Interestingly, since the technique relies on opto-acoustic effects involving the response of the charge carriers, doping profiles can also be extracted [175]. Time-domain CAP investigation thus corresponds to a powerful, non-invasive, experimental tool for the depth profiling of many physical properties with a spatial resolution down to several tens of nanometers. The resolution is here defined by the propagating phonons’ spatial extension, which depends on the launching conditions (see Section 3.1). Remarkably, since probe light scattering preferentially occurs at the sharp fronts of the propagating strain profile, depth resolution can effectively appear smaller than the CAP wave packet extension [164]. Finally, advance mechanical study of wave propagation can be implemented, for instance with the monitoring of acoustic non-linearities inferred from time-dependent frequency and dispersion of the oscillating signals [164].

Damping of the oscillating signal is governed by inelastic relaxation of the CAPs into incoherent phonons and effective dephasing due to elastic scattering at interfaces and inhomogeneities. In addition, effective damping is introduced by the limited penetration depth of the probe beam in a not strictly transparent material. In fact, a significantly different regime for the monitoring of the propagating CAPs is reached when probe light is strongly absorbed. Only a shallow region at the top of the sample is monitored, with little to no oscillation induced in the optical signal. However, acoustics echoes can be observed [147,153,165]. They appear at delays corresponding to the time needed for the CAPs to reach the monitored region, usually after one or several reflections. Investigating echoes brings complementary information on the sound velocity and sample thickness through the trip time delay, and on intrinsic damping and scattering at interfaces through the relative amplitude attenuation. Interestingly, the penetration depth is tunable with the probe wavelength, which translates into a tunable bandwidth for the probing of the CAPs, with highest frequencies probed at shortest penetration depth [155,176].

Thirdly, a resonant regime of coherent phonons is observed when the system is reduced in size to a sub-micron scale, as depicted in Figure 3c. More precisely, it corresponds to the case where the coherent phonon launching region, governed by the direct opto-acoustic interaction and the photocarriers diffusion, is spread along the full size of the material in the direction of phonon propagation. These resonant coherent phonon (RCP) modes can be alternatively described as Raman-active breathing/shear modes or as CAPs, which no longer propagate but rather form a standing wave due to the boundary conditions. They thus present similar features as the ones discussed previously. This regime is intentionally highlighted in this review due to its high relevance in few-layer vdW material studies (see Section 4.3). Such acoustic oscillations have first been observed in thin films with a thickness in the several tens of nanometers range [146]. It corresponds to the simplest one-dimensional case of confinement. In Reference [146], the displacement is in the out-of-plane direction and a perfect contact to the stiffer substrate was obtained. The resulting fundamental resonant acoustic mode presents a spatial wavelength λ=4h, with h being the film thickness, and a corresponding temporal frequency

(5) $$f=\frac{v}{4h}.$$

Further confinement in the other dimensions leads to the case of nanoparticles, for which RCPs have been investigated first in ensemble of semiconducting quantum dots [177] and metallic nanospheres [178,179,180], and more recently at the single nanostructure scale [181,182]. The spatial and temporal description of the RCP modes can be expressed analytically for simple geometries or may require more complex calculation such as finite element modeling [183]. We further emphasize that in this regime the time-domain oscillating signal depends on both material properties and its specific morphology (size and shape).

As most properties of a nanoscale structure, RCP features are strongly influenced by the direct environment of the nanoparticle. The experimental frequency can be significantly modified by a mechanical coupling to a substrate, a matrix, or molecules at the surface [184,185,186]. Inhomogeneous distribution of those vicinity effects, in addition to inhomogeneous morphology distribution, induces effective dephasing of the oscillating signal when probing large area of thin films or ensemble of nanoparticles [180]. When homogeneous quality factors are addressed, mechanical coupling to the environment is usually the dominating source of damping due to the induced loss of energy [187,188]. Hence, well-isolated systems, such as suspended films, are required to investigate the intrinsic sources of damping, related to phonon-phonon scattering mechanisms [189].

A widely utilized hybrid scheme consists in combining a nanoscale thin film, which acts a transducer, with an investigated transparent system. This approach is in direct analogy with the ultrasonic pulse-echo technique on macroscopic system mentioned in Section 2.3, but here with a strikingly higher spatial and temporal resolution. Likewise, a strain wave is emitted as RCPs at the transducer upon pump excitation, it subsequently propagates as a CAP wave in the sample, it gets scattered and reflected at interfaces and inhomogeneities, and finally reaches back the transducer after some time delay, which is monitored by the probe pulse. Usually, metal thin films are used as transducer because of their ease and versatility of deposition on various surfaces, and their strong optical absorption [190,191]. This scheme combines efficient emission of the propagating CAPs even in transparent materials, enhanced detection, high phonon bandwidth, and high depth profiling resolution (from the controlled reduced size of the absorption depth, which directly corresponds to the metal film thickness) [170,192]. Alternatively, acoustic transducers can be made of a quantum well embedded in a crystalline structure. Lattice matching and reduced thickness (down to around 10 nm) further improve response amplitude and frequency bandwidth (up to 1 THz) [193,194]. Interestingly, comprehensive analysis of the optical signal induced by a quantum well transducer yields precise acoustic wavefront reconstruction [195,196]. Finally, building on these considerations, advanced control over the emitted and detected acoustic waves can be developed using superlattices to engineer the effective phonon dispersion [197,198,199].

In conclusion, time-domain investigation of coherent phonons forms a mature, yet still expanding, field of research that has reached a wide range of characterization applications in condensed matter but also in soft matter, porous medium, bio tissues, etc. Applied to vdW materials, it yields the versatile, non-invasive, coherent probing of acoustic and optical phonon modes. This enables, in an original way, the high-resolution profiling of their mechanical properties, especially in the out-of-plane direction. Potential of this approach is explored in the next section through the review of the reported experimental investigations which make use of this original technique.

## 4. Coherent Phonons in vdW Materials

As discussed in the previous section, time-domain investigation is a powerful tool for probing the relaxation of energy between electrons and towards coherent and incoherent phonons. To date, it has been primarily utilized in monolayers, few layers, and heterostructures of vdW materials to explore the dynamics of emerging exotic optoelectronic phenomena. This will be shortly reviewed at first in the following. In contrast, the probing of coherent phonons in time-domain has barely addressed bulk vdW materials, and a stronger interest in thinned down vdW layers only progressively grew during the last few years. However, a large set of configurations have already been explored, in terms of vdW material nature, thickness, synthesis method (exfoliated, CVD, colloidal), with isolated crystals on a substrate, suspended or imbedded in a heterostructure. We propose a comprehensive review of the experimental studies using an all-optical time-domain scheme, organized according to the nature of the probed coherent phonons in echo to the previous discussion in Section 3.2. Other time-domain probe schemes, which involve X-ray or electron monitoring, will be discussed at the end.

### 4.1. Electronic and Thermal Relaxation

The optical probe in time-domain is sensitive to any physical modifications induced by the pulsed pump that would influence the dielectric function of the material in the probed area. The electronic and thermal responses usually are the dominating signals at different timescales from femto- to nanoseconds. Since the interest in thin vdW materials was first driven by their exotic intrinsic electronic properties stemming from the unscreened, 2D confined charge carriers, time-domain investigations have been primarily utilized to explore these effects. The recent advances in this field illustrate the richness of such time-resolved investigations. Here we briefly discuss them and emphasize the peculiar electron-electron and electron-phonon behaviors which can impact the coherent phonon generation.

On ultrafast pico- and sub-picosecond timescales, strong carrier interaction enhanced by the low dimensionality is at the origin of a large set of photocarrier dynamics. For instance, the physics of the room-temperature stable, long-lived excitons in semiconducting TMDs have been largely investigated [200]. Different aspects from the build-up of the excitonic quasiparticles [201,202], to their subsequent diffusion [203], Auger interaction, and other recombination mechanisms [204,205], have been resolved through all-optical time-domain studies. In addition to photocarrier incoherent population, the ultrafast coherent dynamics of specific features (valley in the Brillouin zone, spin…) can be followed, and controlled, using advanced schemes [206,207,208]. Another peculiar case is the one of graphene. Femtosecond electron-electron thermalization in this semimetal yields a hot carrier distribution, with a temperature much higher than the one of the lattice, which drives the physical response after optical pulsed excitation [209]. Probes in time-domain based on optical [210] but also THz radiation [211,212] and photocurrent [213,214,215], are implemented to follow the ultrafast electronic transport and relaxation mechanisms. A comprehensive picture that involves competing scatterings to strongly coupled optical phonons, weakly coupled acoustic ones, remote phonons, and impurities from the environment is depicted [216,217].

Furthermore, the coupling between layers, in particular in heterostructures, can be characterized in time-domain [218]. Ultrafast energy [219] or charge [220,221] transfer depending on the band structure alignment in hybrid layer junctions has been evidenced. Additional cooling pathway can be introduced for carriers in graphene through scattering with hBN hyperbolic phonon polaritons in an encapsulated monolayer [222]. On longer timescales, anisotropic thermal conductivities in bulk and thin vdW films can be investigated [87,88]. Time-domain investigations appear as powerful means to perform characterization and extraction of figures of merit of ultrafast hybrid photodetection schemes in engineered vdW heterostructures [223,224]. Overall, this opens the way for vdW material-based (opto)electronic devices (photodetectors, emitters, modulators…) with exotic features, optimized efficiency, and heat management [225,226,227,228].

**Figure 4:**
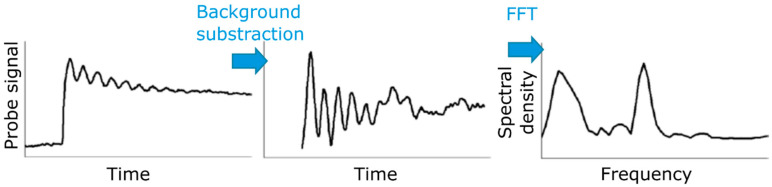
Typical processing of the optical signal measured in time-domain investigations in order to extract the oscillating components related to the coherent phonons, and the corresponding acoustic spectrum. From [137], published by the American Physical Society under Creative Commons.

In comparison, investigation of coherent phonons in vdW systems is still in its infancy, with many basic elements still to uncover. However, the thorough knowledge on relaxation mechanisms developed over the years is of high relevance to find optimized conditions for coherent phonon excitation and detection, and to accurately remove electronic and thermal responses in the monitored optical signal. Isolation of the time-domain coherent phonon signal in reported investigations mostly consists in empirical removal of the signal non-oscillating component, as described in Figure 4. Ultrafast build-up related to electronic response may be ignored or unresolved, and an effective fit, with single exponential or more complex function, is used to reproduce the slow decay related to further internal and external relaxations. Quantitative discussion on the generated coherent phonons is based on the remaining oscillating signal using a fit with one or several damped sine waves and/or spectral analysis via fast Fourier transform (FFT).

### 4.2. Coherent Optical Modes

We first bring our focus on the periodic signals attributed to coherent *optical* phonons. Such oscillation is characterized by a high frequency, i.e., typically around several THz, which is almost independent on the number of layers and on the pump and probe wavelengths, and with a relatively high quality factor, around several 10s up to more than 100. It is thus characteristic of a bulk-like signal, localized in the probed area, driven by the lattice properties and not, or barely, by the dimension and specificities of the sample. It has been observed in vdW materials under different forms: Bulk [229,230,231], exfoliated thick [39,142,232,233,234,235] and thin [133,139,236,237,238,239,240,241] slabs as well as colloidal sheets [242].

**Figure 5:**
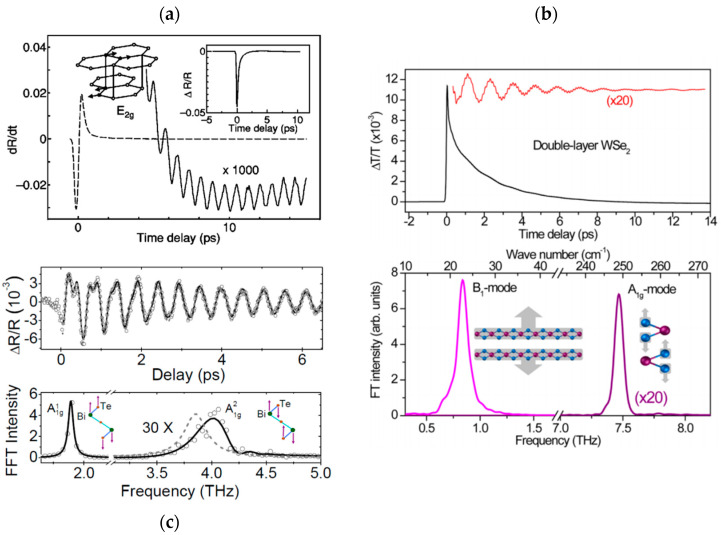
(**a**) Time-domain optical signal, here transient reflectance, measured in bulk graphite, with extraction of oscillations related to coherent optical phonons (COPs). Reprinted with permission from [229], Copyright ©2000 by the American Physical Society. (**b**) Time-domain optical signal, here (top) transient transmittance and (bottom) corresponding spectrum, measured in bilayer WSe_2_, with extraction of both coherent optical and resonant phonons. Reprinted with permission from [133], Copyright ©2016, American Chemical Society. (**c**) Time-domain optical signal, here (top) transient reflectance and (bottom) corresponding spectrum, measured in Bi_2_Te_3_. Reprinted with permission from [233], Copyright © EPLA, 2010. Schematics of the respective modes are presented in insets.

The identification of the specific optical phonon modes at the origin of the oscillations is usually based on the comparison with the modes extracted from experimental Raman spectroscopy and ab initio calculations. In the case of bulk graphite as in Figure 5a, E_2g1_ (i.e., interlayer shear optical mode at 1.3THz) and E_2g2_ (i.e., in-plane intralayer stretching mode, related to the well-known Raman G band at 48 THz) have been identified [229,230,231,236]. In the case of three- and five-atom-thick chalcogenides, such as WSe_2_ (Figure 5b) [133], WTe_2_ [232], and SnSe_2_ [242] for the former, and such as Bi_2_Te_3_ (Figure 5c) [139,233,238,241], Bi_2_Se_3_ [39], and Sb_2_Te_3_ [234,235,237,240] for the latter, the experimentally observed main oscillation is attributed to the A_1g1_ mode, an optical mode at a few THz that consists in the intralayer out-of-plane oscillation of the atoms at the top and bottom of each layer, in opposition of phase. In several studies on QLs, a weaker peak at higher energy can be observed, attributed at first to second harmonic generation [238] and consistently related to the A_1g2_ mode afterwards [39,233,241].

By varying experimental parameters such as pump and probe beam polarization, fluence, and wavelength, generation and detection mechanisms can be investigated, bringing at the same time further information on the involved phonons. For instance, in graphite under infrared light, the electronic and COP optical signals’ amplitudes share a similar linear increase and saturation with fluence. This indicates that the same excitation scheme is involved, namely a real excitation of π-π* bands transition is at the origin of the emission of the COPs [229,231]. In metal dichalcogenides, higher oscillation amplitude is found when probe light is polarized along a specific crystal axis, in agreement with the A_1g_ mode symmetry [232], and when pump light wavelength is close to the excitonic absorption resonance, indicating that a displacive COP generation mechanism is involved [133,232]. Noticeably, calculations show that electronic bandshifts are more sensitive to the displacement of A_1g_ than E_2g_ modes in metal dichalcogenides [133]. This can explain why only the former is observed in a pump-probe experiment while both are Raman active with a similar amplitude. Interestingly, calculations based on molecular dynamics allow discussion about the phonon–phonon couplings (coherent A_1g_ modes with other intralayer optical phonons and interlayer shear modes) to describe the generation and dissipation mechanisms of the coherent signal in QLs [241]. The main generation process in these structures is identified as driven by a photo-induced stress through either thermoelastic effect [241] or deformation potential [139], illustrating that discussion is still open. Overall, we find that a time-domain investigation of optical phonons in vdW materials is complementary to the more usual Raman spectroscopy due to the different physical mechanisms at play. Furthermore, a time-domain study can take advantage of the coherent nature of the phonons by following the phase of the oscillating optical signal. In this context, the phase at start in metal dichalcogenides corresponds to the one of a cosine wave, which further confirms the identification of a displacive generation mechanism [242].

The study of the COPs allows the overall probing of the lattice properties, in a complementary manner to Raman and other structural probes. The anharmonicity of the modes has been investigated in chalcogenides [133,232,233,239] and graphite [229] through the softening of the modes with a rising temperature. In addition, a direct correlation between the damping of the oscillations in the optical signal and the defect density has been demonstrated in graphite [230] and in QLs stacks [239]. A stronger damping is also generally observed in natural QLs superlattices [237]. In these structures where intercalation of individual atoms or other vdW layers is induced by a change in the growth stoichiometry, a strain-induced shift of the coherent modes can be resolved as compared to the expected frequencies from a homogeneous stack [39]. When lowering the number of layers below typically 10, the observed optical modes show a softening compared to the value in the bulk form, which is slight for out-of-plane A_1g_ mode in WSe_2_ [133] and more pronounced for shear E_2g1_ mode in graphene [236]. In the latter case, similar changes with the number of layers in the oscillation frequency have been extracted for suspended, supported, and capped few-layer crystals, which supports an intrinsic origin rather than environmental effects.

In a strong perturbation regime, pump-probe experiments enable the exploration of ultrafast far-from-equilibrium behaviors, given that no material damage is introduced [240]. Under high pump fluence, stronger damping of the A_1g_ coherent phonons is observed, which is attributed to the increased photo-excited electron population [232,237,238]. Furthermore, evidence of non-linear lattice dynamics is demonstrated in WTe_2_ [232] by a softening the coherent mode, as it can be expected in conventional materials. Conversely, an exceptional stiffening and lower dephasing rate is observed at high fluence in graphite for the E_2g2_ mode [231]. This stiffening is also obtained at short timescales, where the experimental dynamically extracted frequency is first upshifted and afterwards converges within a few picoseconds towards the value extracted from Raman spectroscopy. This is understood by considering the out-of-equilibrium electron distribution and its non-adiabatic coupling to the lattice [231]. In QLs, time-dependent study of the frequency of the A_1g1_ and A_1g2_ coherent modes shows a downshift at short timescale for the former, and a linear chirp for the latter [233]. The prevalence of these effects at high fluence and low temperature (where carrier diffusion is reduced) points to an influence of the photo-excited carrier density, though the exact mechanism is not fully understood. In this context, time-domain studies provide an original probe of the electron-electron and electron–phonon interactions under out-of-equilibrium regimes, which are of high interest in low dimensional materials such as vdW layers.

**Figure 6:**
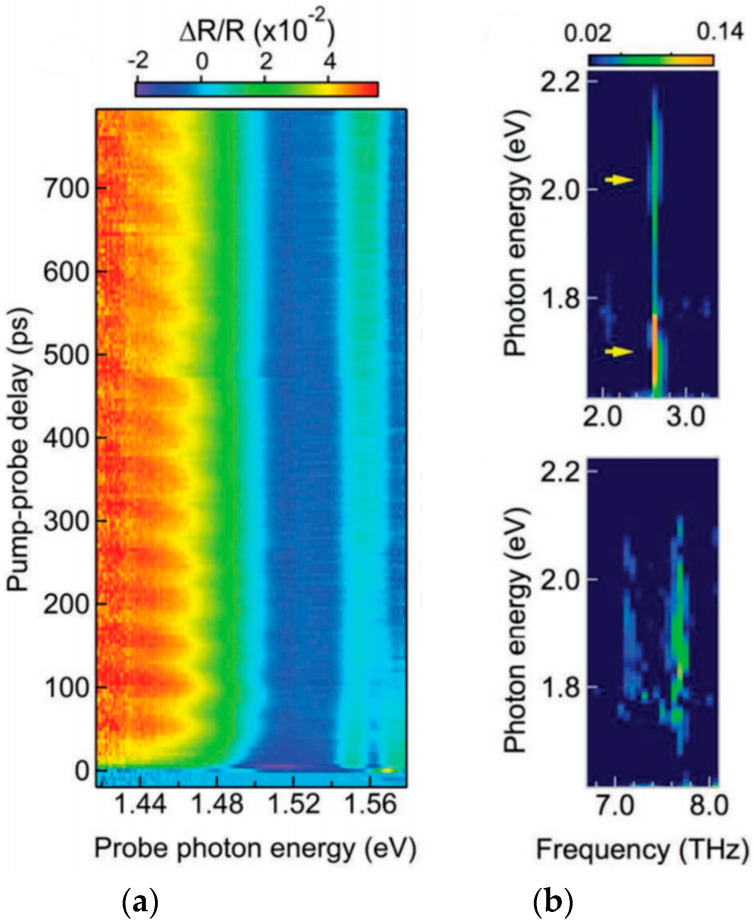
(**a**) Time-domain optical signal measured in Re_6_Se_8_Cl_2_ as function of time and probe photon energy. Oscillations in the THz (attributed to COPs) and in the GHz (attributed to propagative coherent acoustic phonons (CAPs)) are respectively observed for energies above and below the optical bandgap at 1.50 eV. (**b**) Intensity of the corresponding spectrum for the (top) 2D in-plane and (bottom) 0D cluster breathing modes showing different resonances related to different opto-acoustic mechanisms. Reprinted with permission from [142], © 2019 WILEY-VCH Verlag GmbH & Co. KGaA.

Finally, a time-domain study in Re_6_Se_8_Cl_2_ layers exemplifies the investigation of different optical modes from their frequency and generation process (Figure 6) [142]. This material presents a singular layered vdW structure of 2D sheets composed of 0D clusters. Accordingly, two types of modes with different range of frequencies can be observed corresponding to the different dimensionalities: 0D breathing modes of the clusters at high frequencies and 2D in-plane delocalized inter-superatom modes at lower frequencies. The formers are characterized by similar polarization selection rules as their counterpart in Raman spectroscopy and by a flat amplitude as function of the probe wavelength. The optical signal in the time-domain study is therefore defined by the Raman tensor, and corresponds to a generation through impulsive Raman excitation. Conversely, a higher amplitude is observed for the latter frequency when probe light is in resonance with the electronic transitions, this mode corresponding to a displacive generation mechanism.

### 4.3. Propagative Coherent Acoustic Phonons

**Figure 7:**
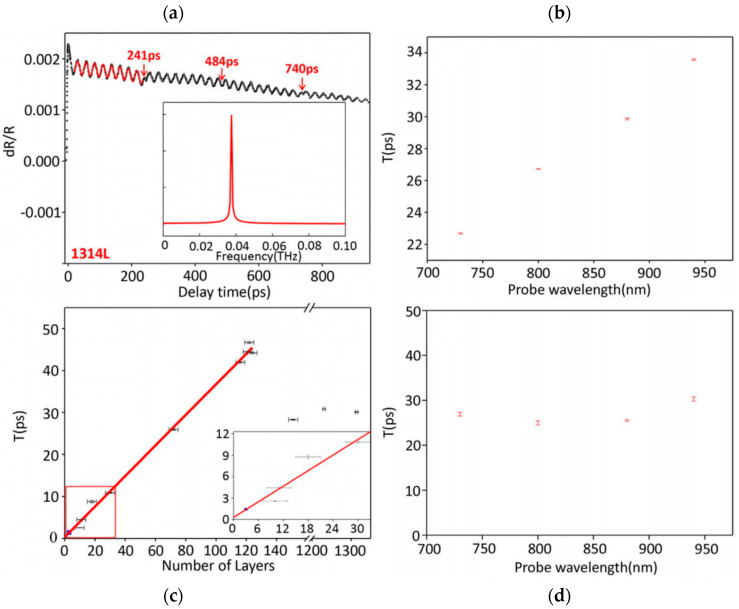
(**a**) Time-domain optical signal measured on a thick sample (around 800nm) of MoS_2_ with oscillations attributed to propagative CAPs with phase shifts at single trip delays. (**b**) Wavelength dependence of the extracted oscillation period in the same thick sample, which follows Equation (4). (**c**) Oscillation periods extracted for samples with different thicknesses. Two regimes are observed corresponding to propagative CAP for thick samples and resonant coherent phonon (RCP) for thin ones. (**d**) Wavelength dependence of the extracted oscillation period for a 44-nm-thick sample, in the resonant regime which follows Equation (9). Adapted from [143], published by Springer Nature under Creative Commons.

In contrast to COPs, the generation of coherent *acoustic* phonons induces the propagation of a strain in the material, in particular along the out-of-plane direction in a thick vdW material. It is the case when the thickness is larger than the pump pulse penetration depth: Light absorption is limited at the interface and yields a localized stress, which induces a strain wave, which can then propagate further inside the material. The probe beam is scattered at the propagating CAP wave front yielding a time-oscillating optical signal, given that the optical wavelength in the material is smaller than the film thickness. As discussed in more details in Section 3.2 with Equation (4), this Brillouin oscillation frequency for a beam collinear with the propagation direction follows the simple expression f = 2n_pr_v/λ_pr_. The frequency depends linearly on the inverse of the probe wavelength and lies in the tens of GHz range, as shown in Figure 7b, in strong contrast to the previously reviewed COPs. Such phenomenon has been observed in thick exfoliated Re_6_Se_8_Cl_2_ (see Figure 6) [142], BP [125], MoS_2_ (see Figure 7) [143], Bi_2_Se_3_ [39,235], and Bi_2_Te_3_ [138], which all are semiconducting vdW materials with interestingly different 2D lattice structures. In these examples, samples are pumped above their respective bandgap yielding pump penetration depths evaluated in the 100-nanometers range or below, and significantly smaller than the slab thicknesses.

From the optical oscillations’ frequencies, material parameters can be extracted such as the acoustic sound velocity for the involved modes and the optical index at the probe wavelength. Extracted out-of-plane longitudinal velocities v_B_ are reported in Table 1 and discussed in more details in the next section. We note that, since the product n_pr_v is effectively extracted in the collinear beams configuration, the value of one of the two parameters must be assumed or independently measured. Therefore, incorrect evaluation of one parameter affects the other. In this context, due to incorrect assignments of echo instead of single trip delays in the oscillation (see below) and of the corresponding resonant acoustic mode (see Section 4.4), a value twice higher than expected for v and thus one twice lower for n_pr_, are reported in MoS_2_ in Reference [143]. Interestingly, the optical anisotropy in BP leads to slightly different optical indices for orthogonal probe polarizations, which induces different Brillouin frequencies even though the same propagating CAP with a single given velocity is probed [125].

Since CAPs propagate in the material away from the top interface, acoustic reflection and transmission at the bottom interface influence their further propagation, and hence the optical signal after some time delay directly related to the sound velocity and sample thickness. Importantly, the observed damping of the optical signal does not directly correspond to the one of the acoustic phonons. In the case where the material is transparent at the probe wavelength, namely below the bandgap for semiconducting materials [142,143], the propagating CAPs are optically followed in the whole structure and a change in phase and/or amplitude is observed in the optical signal after the CAP wave reaches the bottom interface, with a time delay corresponding to a single trip along the film thickness, as presented in Figure 7a. Both transmitted (in a different material) and reflected acoustic waves are probed afterwards, leading to an effective dephasing of the optical signal faster than the intrinsic damping of the CAPs. Conversely, if the probe wavelength lies above the bandgap, the probe penetration depth becomes lower than the film thickness and therefore drives the effective damping of the optical signal oscillations even though the phonon behavior remains unchanged [39,125,235]. In this regime, different effective quality factors are reported for different probe polarizations in BP due to its optical absorption anisotropy [125]. Moreover, echoes are observed when the propagating CAPs bounce back and forth inside the slab, at time delays corresponding to twice the trip over the film thickness [125,138]. When comparing amplitude reduction of such successive echoes, stronger damping in natural superlattices of QLs is found compared to bulk stacks [138].

Finally, the CAP generation mechanisms can be investigated using similar experimental means as the ones reviewed in the previous section about COPs. For instance, from the oscillation phase at the start and the pump polarization dependence, a direct deformation potential mechanism has been reported for the launching of CAPs in BP [125].

### 4.4. Resonant Coherent Phonons

One of the specificities of vdW materials is that they can be thinned down to the atomic scale without introduction of structural defects. The regime of *resonant* coherent phonons reached for thin slabs has thus caught most of the attention in the reported time-domain experiments. These oscillations are referred to as nanomechanical resonances, (inter)layer breathing modes, or standing (sound) wave modes. They are characterized by an extracted frequency in the GHz range, which no longer depends on the probe wavelength, but increases when the material gets thinner, as shown in Figure 7c,d. This has been observed in exfoliated few layer crystals of WSe_2_ [133], MoS_2_ [143], MoSe_2_ [132], InSe [135,136,137], PtSe_2_ [134], BP [126], Bi_2_Se_3_ [243], and Bi_2_Te_3_ [139]. In all these examples, the penetration depth of the pump beam gets larger than the thickness of the sample. This leads to a homogeneous optical excitation of the slab. The transition between regimes of propagative CAPs and such resonant modes, presented in Figure 7c, was observed for MoS_2_ at around 120 layers (75 nm) in agreement with the absorption depth of the 400 nm pump wavelength [143]. We note that the sample thickness and corresponding number of layers are experimentally evaluated in the reviewed investigations from atomic force microscopy, and at times corroborated with Raman spectroscopy.

The RCP modes can be first described from their spatial wavelength λ, which is defined by nodes or anti-nodes at each interface. In the following, we consider only the cases where the slab is uncapped. The absence of an overlay implies that an anti-node is found at the top interface. We thus have

(6) $$\lambda =\frac{4\mathrm{Nd}}{2j-1}\;\text{with a node at the bottom},\;\text{and}$$

(7) $$\lambda =\frac{2\mathrm{Nd}}{j}\;\text{with an anti-node at the bottom}.$$

where N is the number of layers separated by a distance d and j is a positive integer lower than N, i.e., 1≤j≤N−1, corresponding to the mode index. The fundamental spatial waveforms when j=1 are presented in Figure 3d,e for the two cases. This yields frequencies

(8) $$f=\frac{v}{\lambda }=\frac{\left(2j-1\right)v}{4\mathrm{Nd}}\;\text{with a node at the bottom},\;\text{and}$$

(9) $$f=\frac{\mathrm{jv}}{2\mathrm{Nd}}\;\text{with an anti-node at the bottom}.$$

**Figure 8:**
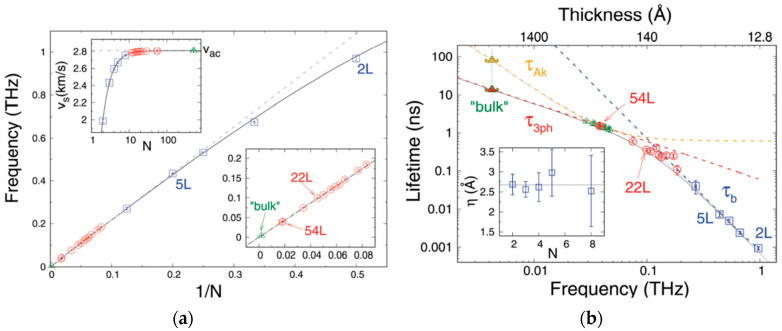
(**a**) RCP frequency extracted from time-domain measurements on suspended MoSe_2_ slabs. Evolution with the number of layers diverge from Equation (9) shown in dashed line to follow Equation (2) shown in solid line for few-layer samples. Extracted acoustic velocity is presented in top inset. (**b**) Corresponding lifetime which follow two different regimes discussed in the main text. Inset shows the average asperity size at the surface extracted from fit to few-layer data. Republished with permission of Royal Society of Chemistry, from [132] Copyright ©2019. Permission conveyed through Copyright Clearance Center, Inc.

For a given mode, the linear dependence between the frequency and the inverse of the thickness of the crystal is experimentally well verified in the reviewed papers. We note that the presented simple linear expressions for the mode frequency are valid in the continuous medium limit, i.e., 2πd/λ<<1, where the bulk acoustic sound velocity can be properly introduced (see Section 2.4). This limit can interestingly be expressed as j<<N for our layered case. This condition is obviously not verified when vdW crystals are reduced to only a few layers. Deviation from the simple linear dependence has thus to be considered to reproduce experimental data, and Equation (2) computed in the rigid-layer framework in Section 2.4 has to be used, as presented in Figure 8a [132]. Furthermore, although only the fundamental mode is usually observed in experiments, higher harmonics can also be probed [136], which reduces even more the range of validity of the continuous medium expressions.

In the different experimental examples in the literature of deposited thin flakes without any capping layer, extracted frequencies always fit one of the two configurations, with either a node or an anti-node at the bottom interface. This yields the quantitative evaluation of the out-of-plane longitudinal (i.e., breathing) acoustic velocity v_B_ for the large set of studied vdW materials. They are reported in Table 1 along with mechanical parameters discussed in Section 2 and values from propagating CAPs from Section 4.3. We find an overall very good agreement for all materials where comparison can be done, with variations usually below 5%, in the same range as of the variability already encountered in the various samples and techniques reported. We mention that the out-of-plane transverse (i.e., shear) acoustic velocity is only extracted for the clustered layers of Re_6_Se_8_Cl_2_ [142]. Notably, v_B_ and the other corresponding mechanical parameters in this complex layered system still fall in the usual range for vdW materials. The velocity of propagating CAPs in thick Bi_2_Se_3_ shows only slight variations when atoms or other type of vdW layers are intercalated, in contrast to COP frequencies [39]. A large variability is found for BP between values reported in literature [127,128,129] and the ones extracted from propagative [125] and resonant [126] modes. Although this can originate from sample and technique uncertainties, the lower values of v_B_ extracted for thinner slabs might originate from boundary effects, which dominate only for few-layer samples. This behavior is clearly evidenced in MoSe_2_ for which the extracted acoustic velocity shows a gradual reduction for decreasing number of layers (starting below 10 layers, see Figure 8a) from 2820 m/s in bulk crystal down to 2000 m/s in a two-layer film [132]. Velocity in thin Bi_2_Se_3_ is similarly reduced from 3000 m/s in bulk and 40-nm-thick stacks down to 2000 m/s for a thickness of 15 nm [243]. This is interpreted as a progressive coupling with shear modes (which are softer than breathing modes) for thinner slabs. For even thinner crystals, RCP signal fully disappears. This is explained by coupling and thus energy loss to flexural and dilatation in-plane modes, which are not optically active. The predominance of this effect in such materials might be related to the relatively large thickness of the layers compared to other vdW materials, around 1 nm in QLs [243].

The assignment to the node/anti-node case for a given experimental configuration consists in a good characterization of the nature of the interface. Though it is obvious that a fully suspended film yields anti-nodes at both ends [132], the assignment when the crystal is lying on a substrate is not as straightforward. The hypothesis in early studies was to assume a node when the layer is supported. This could lead to wrong assignment and error in the values of extracted parameters (see [143], commented in [135] and Section 4.3). It was later acknowledged that a *bad* microscopic contact between the deposited vdW crystal and the substrate could induce an effective decoupling of the mechanical vibrations, and the presence of an anti-node at the interface [133,135]. Noteworthily, even a *good* mechanical contact can yield an anti-node if the substrate has a low rigidity, for instance polymers [126]. This issue can be quantitatively discussed by introducing the acoustic impedance in the rigid-layer model [137,184,244]

(10) $$Z={\rho v}_{B}=\sqrt{K\mu }.$$

Values for the reviewed vdW materials and substrates are reported in Table 1. We note that, in a similar way as for other vdW mechanical parameters, Z spans a relatively small range of values, from 7 to 23 MPa.s/m. The impedance mismatch between the vdW film (of thickness Nd and of impedance Z_f_) and the substrate (semi-infinite, of impedance Z_s_) drives the interface mechanical behavior. Solving this geometrical configuration in a continuous medium framework, one finds that the complex frequency $\tilde{f}$ of the acoustic oscillation in the thin film is solution of the equation [245,246,247]

(11) $$\exp\left(4i\pi \tilde{f}Nd/v\right)=\left(Z_{f}-Z_{s}\right)/\left(Z_{f}+Z_{s}\right)=R,$$

where R is the acoustic amplitude reflection coefficient. The analytical solution presented in Equation (8) (Equation (9)) corresponding to a node (anti-node) at the interface is obtained for the real part of $\tilde{f}$ when Z_s_ > Z_f_ (Z_s_ < Z_f_). Only limit cases when Z_s_ >> Z_f_ or Z_s_ << Z_f_ strictly correspond to stationary resonant modes. For intermediate cases, general Equation (11) introduces a damping of the oscillations with a non-zero imaginary part of $\tilde{f}$, related to energy loss to the substrate, which increases as Z_s_ and Z_f_ values come closer together. Consequently, close impedance values between the two materials yield strong radiative damping, which hinders the resonant oscillation. Following this consideration, materials such as amorphous glass, quartz, or silicon are not recommended substrates for usual vdW materials [136,139], but stiffer materials should be preferred such as sapphire.

**Figure 9:**
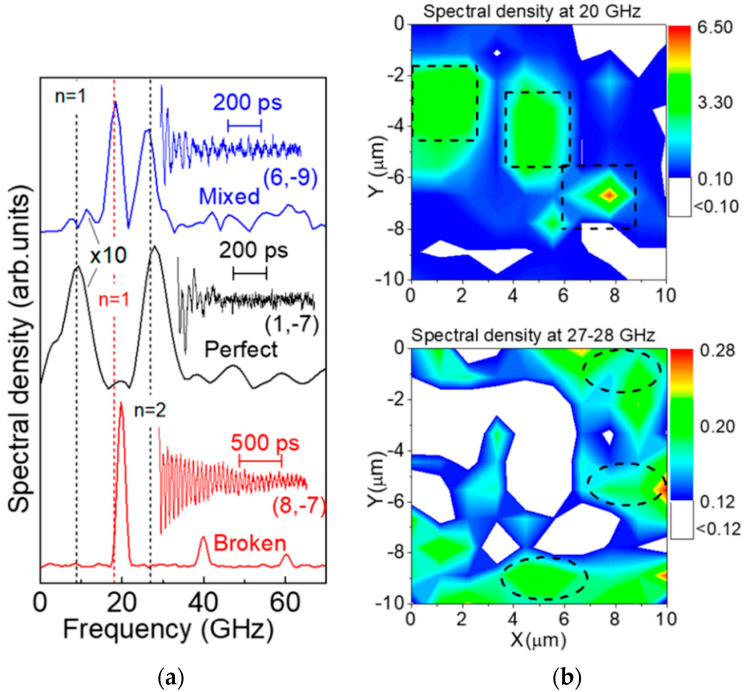
(**a**) Spectral density extracted from time-domain signals (shown as insets) measured on different areas of a 60-nm-thick InSe crystal. (**b**) Integrated spectral density over frequency ranges corresponding to (top) bad contact to the substrate and a match to Equation (9) with j = 1 or (bottom) good contact and a match to Equation (8) with j = 2. Republished with permission from [136], Copyright ©2019, American Chemical Society.

Going further, Greener et al. [137] introduces a model considering a non-perfect contact with a fitted stiffness at the substrate/vdW film interface, analogue to the interlayer elastic constant K in the vdW material. This slightly modifies the modelled resonant frequencies and allows better fitting of the experimental signals. Extracted values for the contact stiffness span from no coupling to a typical vdW bond. Interestingly, intermediate stiffness values introduce a frequency dependence for the contact property, which is not the case in the ideal acoustic mismatch framework. A contact appears as *good* (*bad*) for film oscillation frequencies below (above) the characteristic frequency of this newly introduced substrate/film elastic constant [137]. Spatial heterogeneity of the contact can be investigated from the scanning of the optical pump/probe beams at the surface of the sample, as demonstrated in Figure 9 [136]. Both *good* and *bad* contacts to the substrate can be identified on a single deposited flake with a micron-scale resolution. Complementary Raman spectroscopy and atomic force microscopy were used to further support this analysis. We note here the importance of having a smaller size for the probe beam as compared to the inhomogeneities, which would otherwise lead to the observation of frequencies from both cases (anti-node/*bad* contact and node/*good* contact with a higher impedance substrate) on a single measurement [134].

The nature of the interface can also be investigated through the damping of the oscillations. The case when both *good* and *bad* contacts are identified on a single deposited flake (see Figure 9) allows for the quantitative comparison of the corresponding values of Q all things being equal. Typical extracted values range from below 10 for the former case, to more than 10 up to several dozens for the latter case [136,137]. Thus, a significantly higher Q is consistently observed in the case with decoupled interfaces. This gives evidence for a damping driven by the losses of mechanical energy to the substrate. Such damping contribution can be quantitatively evaluated using Equation (11). It yields

(12) τ=1/(2π Im(f˜))=−2Nd/(v ln|R|),

where R is defined as previously in the impedance mismatch model [246,247] or modified by the interface contact stiffness for an imperfect contact [137]. Remarkably, the damping is independent of the mode frequency, implying that higher Q are expected for higher harmonics, in good agreement with experimental data from [136], which strengthens this interpretation in this recent study. However, a quantitative discussion indicates that energy transfer to the substrate is not the only limiting mechanism [137], which brings the focus on other extrinsic sources of damping.

Significantly lower values of Q have been reported in other studies, even when the vibration frequency agrees with an anti-node at both interfaces and thus with a decoupled flake, with Q≈10 down to a few unities [133,134,135,143,243]. Furthermore, for samples from the same vdW material with similar thicknesses, different Q can be observed [135,143]. In those cases, it appears that inhomogeneous broadening may be the physical phenomenon limiting the experimental Q [137]. Variation of the film thickness due to surface wrinkling, change in the interlayer bond properties, presence of adsorbates at the interfaces, structural defects such as dislocations, local strain, or vacancies might inhomogeneously shift the RCP mode frequency under the probe beam spot and lead to an effectively damped acoustic oscillation. For supported flakes with good contact to the substrate, spatial variation of the interface contact stiffness can similarly introduce effective dephasing effects.

To reach more intrinsic quality factor limitations, measurements on fully suspended crystals must be performed. In this view, Soubelet et al. [132] reported the highest Q, up to around 200, in thin vdW materials by investigating MoSe_2_ flakes, from 2 to 54 layers, exfoliated on top of a substrate with micron-size holes. Two distinct regimes depending on the flake thickness are demonstrated (see Figure 8b) and discussed. For films larger than around 20 layers (14 nm), Q reaches an upper limit around a value of 200. This behavior stems from an intrinsic damping determined by anharmonicity ruling the interaction of the RCPs with other thermal phonons in the crystal. For thin slabs, typically below 20 layers, Q decreases when the number of layers is reduced. This relates to boundary diffusive scattering effects, which become preponderant in such low dimensional systems. More precisely, Q scale with f^−2^, in agreement with a damping mechanism attributed to imperfect reflection of the acoustic modes on asperities at the free interfaces. Average asperity size is fitted to 0.26 nm to match the experimental acoustic data, which corresponds well to adsorbates or structurally induced local variations of the flake thickness. Similar discussion is provided in Greener et al. [137] with the conclusion that this surface damping mechanism is not the limiting one for their relatively thick samples, in the tens of nanometers thickness.

The mechanisms for RCP generation are generally not the focus of the reviewed studies and are thus usually assumed, as thermoelastic [135,143] and displacive [132]. In Bi_2_Te_3_, quantitative calculations indicate a prevailing contribution of the deformation potential mechanism over the thermoelastic one [139]. Additionally, experimental approaches tuning polarization [134], fluence [143], and wavelength [126] of the pump and probe beams can yield interesting insights on the origin of RCPs, in a similar way as already discussed for COPs and CAPs. In addition to yielding a better general understanding of light–matter interaction in vdW materials, such studies are of high importance for enhancing the experimental optical signal, which can be a strong limitation when probing these systems reduced to a size of a few atomic layers.

### 4.5. Van der Waals Transducers

Coherent phonons generation can be efficiently improved by the use of opto-acoustics *transducers*. The principle, already introduced in Section 3.2, consists in using a different system to interact with the pump and/or probe light beams in a more controlled, optimized way than if it were directly with the material under study. Discussion about this scheme naturally lays its foundations in previously introduced points, about propagative over resonant coherent modes and the nature of the mechanical interface between materials.

A first approach is to combine a conventional transducer, namely a metal thin film, with a vdW material that would otherwise be transparent to the pump beam. This was reported in the case of a 30-nm-thick Al film deposited via e-beam evaporation on top of InSe and hBN flakes with various thicknesses [137]. Absorption of the pump beam occurs in the Al transducers, yielding resonant acoustic modes that can further propagate into the underlying vdW material, owing to the fact that good contact and impedance matching is achieved at the interface. Experimental results clearly validate those assumptions. We note that this approach enabled the launching and probing of RCPs in few-layers vdW crystals that might be too thin to yield strong enough interaction with light on their own.

**Figure 10:**
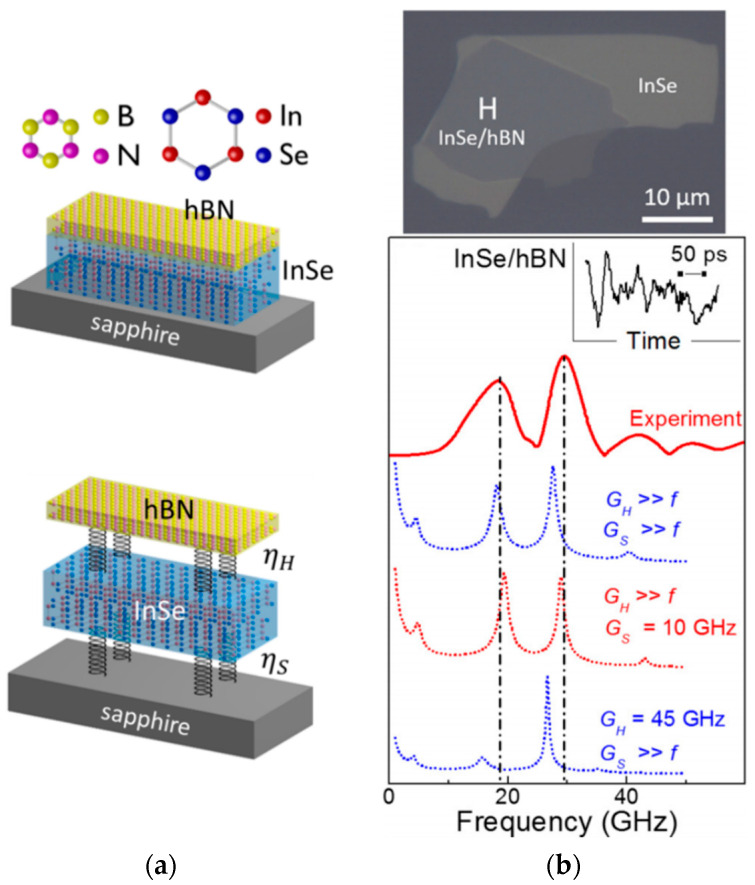
(**a**) Schematic representation of a stack of hexagonal boron nitride (hBN) and InSe crystal on top of sapphire substrate, with introduction of imperfect contacts defined by fitted stiffness. (**b**) (top) Optical micrograph of the stack and (bottom) corresponding spectral density in solid red line extracted from the time-domain measurement in inset. Models in dashed lines find a better match with data in the case of perfect hBN/InSe contact and relatively bad contact with substrate (red dashed line). From [137], published by the American Physical Society under Creative Commons.

Another, eventually more fruitful, approach would be to use a vdW layer as a transducer optically resonant with the pump and/or probe beams, which is directly embedded in a vdW heterostructure where the CAPs propagate. Such approach implies that a good mechanical coupling is achieved at the vdW interfaces. This point was experimentally demonstrated in stacks of InSe on top of a hBN or another InSe crystal [137]. In the case of the InSe/InSe homogeneous junction, the overall vdW stack mechanically behaves as a single material and show RCP mode frequency values in agreement with the full thickness of the heterostructure. This relies on perfect impedance matching. In the InSe/hBN heterojunction presented in Figure 10, RCP modes from both acoustically mismatched films are observed. This however indicates good mechanical coupling and efficient acoustic energy transfer from InSe to hBN since the latter is transparent at the pump wavelength and is not optically excited. In natural superlattices formed by QLs, effective velocities for the propagative CAPs have been experimentally extracted for different spatial periods [138]. They can be compared to the expression obtained in the acoustic mismatch framework for superlattices, which includes the different impedances and thicknesses of the layer sequence [138,244]. A deviation from this model, even stronger when comparing the relative amplitudes, is clearly identified. A more refined model that considers the much larger acoustic wavelength compared to the periodicity of the stack has to be developed. While not perfectly theoretically described, these important preliminary observations validate the feasibility of making use of an engineered vdW heterostructure for a time-domain study.

**Figure 11:**
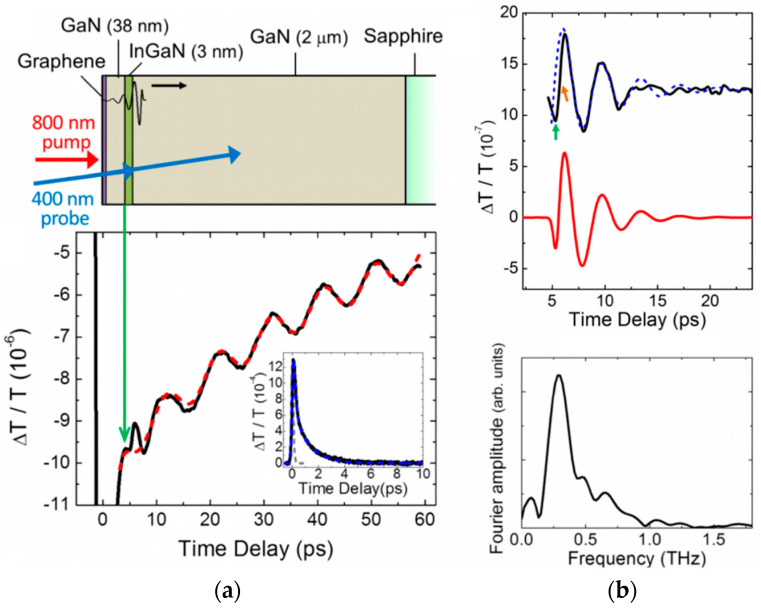
(**a**) (top) Schematic representation of a graphene monolayer deposited on a GaN/InGaN/GaN quantum well heterostructure. (bottom) Corresponding time-domain signal in black whose oscillating components are dominated by a Brillouin oscillation, evidenced as a dashed red line, related to the propagative CAP wave. (**b**) (top) In black, transient signal at the quantum well (indicated by a green arrow in (**a**)) after removal of the previously identified Brillouin oscillation revealing the CAP waveform. Modeled waveform, see main text for details, in red slightly differs from a simple damped oscillation presented as a dashed blue line. (bottom) Corresponding spectrum. Reprinted with permission from [248]. Copyright ©2014, American Chemical Society.

The use of a thin vdW slab as a transducer to launch CAPs in a bulk material can be discussed. In this scheme, an impedance matching must be sought to achieve good transmission of acoustic energy to the bulk substrate. We note that this specific configuration when RCP are hindered, as mentioned in Section 4.4, can be correlated with the observation of Brillouin oscillation in the substrate as shown for Bi_2_Te_3_ on (100)Si [139]. Interestingly, this was also studied in a pioneer work by Chen et al. [248] using a monolayer as transducer. Although the study focusses on the energy loss of a single CVD graphene layer to its substrate via the emission of CAPs, it gives important insights for the use of graphene as a transducer in time-domain measurements. Upon near-infrared pulsed excitation, the launching of CAPs at the interface between a single graphene layer and its supporting transparent substrate, GaN or SiO_2_, and their subsequent propagation are demonstrated. Furthermore, the use of a 3-nm InGaN quantum well embedded in the GaN substrate allows for the spatial waveform reconstruction of the propagative CAP pulse, as presented in Figure 11. The results are understood in terms of a two-steps dilatation and compression mechanism at the graphene/substrate interface [248]. In the first half picosecond, electronic stress associated to photo-excited carriers in graphene induces a dilatation strain at the substrate surface. This is followed, in the next few picoseconds, by thermal stress associated to lattice heating, which corresponds to an expansion of the bonds at the graphene/substrate interface and leads to a compression strain in the substrate. The acoustic pulse can afterwards propagate. Spectral analysis shows that frequencies up to 1 THz are launched, with a resonant mode at 240 GHz. This latter value corresponds to a 2D stiffness K of 2.4 × 10^19^ N/m^3^ and thus relates to the vdW nature of the interface between graphene and the GaN substrate [248]. A strong damping of the coherent oscillations at the interface occurs within a few picoseconds. This appears faster than expected from a perfect elastic contact and mechanisms at play are further discussed by the authors [248].

### 4.6. Non-Optical Probes

**Figure 12:**
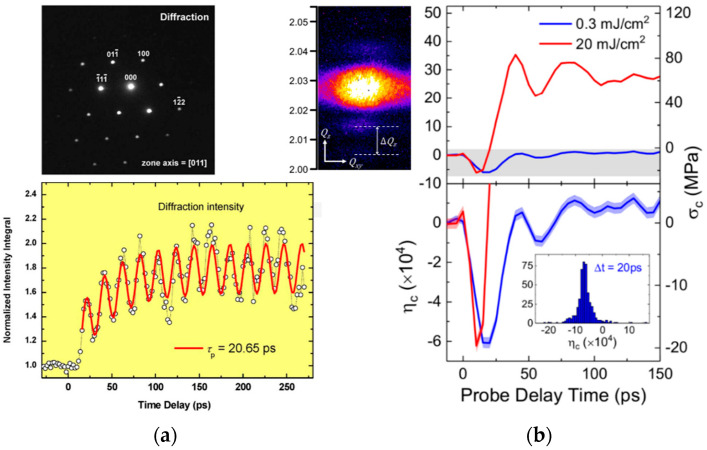
(**a**) Electron diffraction time-domain measurements on 39-nm-thick graphite where (top) diffraction spot intensity yields (bottom) an oscillating signal attributed to a breathing RCP mode. Reprinted from [124], Copyright ©2008, with permission from Elsevier. (**b**). X-ray diffraction time-domain measurements on a 50-nm-thick MoS_2_ slab where (left) the spacing between Bragg spot fringes $\Delta Q_{z}$ yields (right) the transient out-of-plane strain (here $\eta_{c}=\Delta Q_{z}/Q_{z}$) represented for two different fluences. Oscillations corresponds to a breathing RCP mode. Reprinted with permission from [131]. Copyright ©2017, American Chemical Society.

For the sake of exhaustivity and comparison, here we present time-domain investigations that make use of an optical pump and a non-optical delayed pulsed probe formed by either an electron beam or X-ray radiation. This gives a more direct and spatially resolved access to the structural changes while conserving a sub-picosecond coherent probing. In the former case, nanometer resolution is achieved through bright-field imaging. Lattice distortion is followed though the changes in intensity and position of the Bragg spots extracted from selected area diffraction, as shown in Figure 12a [249]. Similarly, X-ray diffraction gives a direct characterization of the structure, and in particular of the interlayer distance in vdW materials as presented in Figure 12b [131]. As compared to previously reviewed all-optical approaches, both techniques can be implemented at the expense of a more demanding experimental setup, more complex sample preparation (larger size, placed under vacuum, etc.) and higher fluence for the pump beam. Consequently, the investigations reported in the following place the probed system very far from its equilibrium, with lattice temperature increase in the range of tens up to hundreds of K, in regimes that might deviate from the one explored in previous sections. In this regime, sp^3^-like bond structure was reported in transiently strongly compressed graphite [250]. VdW crystals can be pushed towards layer ablation [251]. Large yet reversible rippling of monolayers can be induced [252].

As in all-optical schemes, electronic and thermal response are probed in time-domain. In bulk and thin graphite, a compression in the first picoseconds, attributed to the build-up of a hot distribution of thermalized electrons along with a subset of strongly coupled optical phonons, followed by a slow expansion attributed to lattice thermal dilation, are consistently reported [250,251,253]. The former effect is further confirmed with time-domain electron energy loss spectroscopy that evidences, from the increasing of the bulk plasmon spectral intensity, an ultrafast strengthening of the interlayer attraction [254]. This whole picture is in perfect agreement with the well-understood ultrafast graphene dynamics evoked in Section 4.1. Similar electronically induced lattice compression and subsequent thermal expansion is reported in various semiconducting TMDs after above-bandgap pulsed excitation [131]. Interestingly, careful study of the former effect opens a quantitative discussion on the vdW interaction strength as function of the photo-induced increase in carrier density.

Coherent in-plane phonon propagation can be directly followed thanks to the nanoscale spatial resolution in bright field imaging. Applied to thin slabs of TMD such as WSe_2_ [255], TaS_2_ [256] and MoS_2_ [130], in-plane modes are observed at frequencies in the range of a few GHz for antisymmetric flexural modes and a few tens of GHz for symmetric dilatation modes. Corresponding extracted in-plane phonon velocities are in good agreement with known bulk values. Interestingly, the high resolution allows to identify the discrete phonon-nucleation sites in space and time [130,255,256], which correspond to atomic-scale defects and mostly step edges at the layered crystal surface. The stress required to emit the in-plane propagative phonons appears to be provided at nucleation sites by priorly excited uniform out-of-plane oscillations, as evidenced by the picosecond delay before their launching.

Time-oscillating signal extracted from selected area imaging and/or diffraction is attributed to out-of-plane coherent oscillation. In a similar way as for all-optical measurements, different classes of coherent phonons can be probed in such structural time-domain investigations. On very long timescales, a resonant drum-like membrane mode, as introduced in Section 2.3, is observed in graphite thin film with a frequency of around 1 MHz [257]. Coherent optical phonons are also probed, namely the Raman-active interlayer shear E_2g1_ mode at 1.3 THz already discussed in Section 4.2 [251,253]. Finally, periodic signals in the tens and hundreds of GHz observed in thin vdW slabs with a thickness in the tens of nm range are attributed to resonant coherent out-of-plane oscillation, in a similar way as in Section 4.4. When no independent quantitative characterization of the crystal thickness is available, the extracted RCP frequency is used for thickness evaluation by assuming the material phonon velocity from literature [253,256]. In other works, thickness at the probed area is evaluated in situ from the X-ray diffraction Bragg peak satellite distance [131] or from the relative electron energy loss intensity scaled by the scattering mean free path [124,130]. Consequently, phonon velocities for MoS_2_ and graphene can be extracted. They are reported in Table 1. We note that good agreement is obtained for graphite. Significantly lower velocities are obtained for MoS_2_. This can be related to more defective samples (stacking faults, etc.) or to the specific experimental conditions corresponding to high transient temperatures (100s K) where non-linearities might occur.

## 5. Conclusions and Perspectives

Time-domain generation and probing of coherent phonons in vdW materials have recently been demonstrated in the works reviewed in Section 4. Various configurations have been explored in terms of material, sample size, and environment, using optical and non-optical probes. The general coherent phonon modes introduced in Section 3 have been observed, with quantitative description of the extracted frequencies. Time-domain investigation has thus proven to be a powerful technique for mechanical characterization of vdW materials in addition to the ones presented in Section 2. It enables the simple and non-invasive probing of structural properties through the study of both optical and acoustic phonons. It yields information on bulk and interface properties with a high out-of-plane resolution, and probes dynamics on a large span of frequencies up to several THz.

The results in the reviewed studies bring interesting insights about the physics at play in the vdW interaction between the layers. Firstly, acoustic velocities of the longitudinal out-of-plane (i.e., breathing) mode are measured for various materials, in good agreement with theoretical and other experimental structural studies. The values are extracted from propagative modes in thick samples and from resonant modes in thin samples. For very thin slabs, typically below 10 layers, a slight softening of the interaction and a breakdown of the continuous medium limit are evidenced. Additionally, a large effort has been oriented towards the physics at the interfaces. All-optical time-domain investigations offer a quantitative in situ characterization of the interfaces coupling strength and inhomogeneities, even for buried ones. Finally, although reduced in number, a few studies have managed to explore more in-depth fundamental physics of the phonons in vdW materials: Generation mechanisms, far-from-equilibrium behaviors, anharmonicities, and damping pathways have been discussed. However, to date, the experimental issue of interface contamination and inhomogeneity has significantly limited experimental results and fundamental discussions by hindering the reproducibility and the comparison between studies.

Such limitations, which also affected the earliest optical and transport studies in vdW materials as mentioned in the introduction, can be overcome using the current nanofabrication state-of-the-art [8,25,26,27,34]. Large and high-crystallinity flakes can be obtained from exfoliation, chemical exfoliation, or growth approaches, in particular using epitaxy. Transfer on a patterned substrate or other vdW layers with a control over the temperature and/or the atmosphere yields passivated, flat, homogeneous samples. Performing fundamental time-domain investigations on such high-quality vdW systems would enable more thorough characterization of their acoustic properties. In particular, reduction of extrinsic damping at interfaces would yield intrinsically limited oscillations even in the case of few-layer slabs, leading to mechanical resonators in interesting high fQ regimes. Furthermore, the electrical contacting and gating of selected vdW layers can be implemented to induce in situ large tuning of their electronic density, with a strong impact on their optical and electronic properties [18,28]. This would further improve the control and comprehensive study of the coherent phonon generation and/or detection mechanisms.

In addition, the full range of the picosecond ultrasonics advanced schemes can be brought into play. As described in Section 3, investigations that are resolved in pump and probe beams’ polarization, wavelength, angle, and/or shape allow for the study of a significantly larger span of properties than the ones studies in the reviewed works. A non-exhaustive list of examples includes the disentangled extraction of sound velocity and optical index [166,169,170], the generation and probing of acoustic shear modes [171,172], the use of lateral super-resolution [258,259], and the optimization of temporal and out-of-plane spatial resolutions towards precise depth profiling [155,164,176], which may resolve stacking orders in homo- and heterostructures. In sum, combining the state-of-the-art in terms of sample nanofabrication on one side and time-domain acoustics on the other side would open a much wider field of investigation than what has been already studied.

In order to guide and interpret future experimental works, the development of advanced acoustic models has to be considered, beyond the simple rigid-layer model presented in Section 2.4 and used to analyze the reviewed studies. A more general mechanical modeling should consider the interlayer atomic structure and the layer stacking order to further understand the differences between vdW materials. Importantly, modeling of the anharmonicity of the vdW coupling is of high relevance due to the high frequency range and the far-from-equilibrium conditions, which are reached in time-domain experiments. Non-linear regimes deviating from the ones in 3D bulk materials might thus be investigated. Similarly, the coupling between hetero-interfaces might benefit from a more complex modeling. We note that calculations in these directions already exist. For instance, complete spring-mass modeling of several-atom-thick layers have been presented [9,52,119], and advanced approaches such as DFT have been implemented to investigate the vdW coupling [74,75,76], as discussed in Section 2.2. Finally, one can raise the question about the relevance of the acoustic impedance defined in the bulk when analyzing heterostructures reduced to very few or even single layers. This point can be interestingly tackled both experimentally and theoretically.

Since time-domain investigations rely on the interaction between light, electrons, and phonons, the large set of exotic optoelectronic behaviors emerging in thin vdW materials can be taken advantage of to explore original coherent phonons features. As described in Section 3.1, the photocarrier properties, such as lifetime, diffusion, and coupling to phonons are key parameters that drive the generation and evolution of coherent phonons. Due to the strong electron–electron interaction in vdW layers, pulsed excitation can yield original configurations for the photocarriers, such as relatively long-lived hot electron distribution, excitonic quasiparticles, plasmons, phonon-polaritons [200,209], etc. A comprehensive experimental and theoretical knowledge on these exotic physical behaviors upon pulsed optical excitation is well established, with even advanced schemes for coherent control of specific features as discussed in Section 4.1 [206,207,208]. Electron–phonon interaction can be strongly modified by the environment, especially in vdW heterostructures, with the introduction of prominent couplings to remote phonons [61,216,217,222]. Additionally, original phonon ballistic and hydrodynamics regimes can be reached [260,261,262,263]. These considerations open a large span of unexplored routes for investigating and even engineering the emission and detection mechanisms of coherent phonons in thin vdW materials.

Building on the current and future knowledge, the field of van der Waals heterostructures can be further developed as a platform for enhanced acoustics. Overall scheme would consist in separating, and hence optimizing, the absorption, propagation, and detection of coherent phonons in different vdW layers potentially reduced to the atomic scale. This would take deep inspiration from the coherent phonon shaping in III-V heterostructures discussed in Section 3.2 [197,198,199]. This latter case suffers from interface crystallinity and material properties’ variety, which are limited by lattice matching conditions. VdW heterostructures overcome these limitations, with defect-free junctions of atomically thin vdW layer of any nature, and can bring the existing architectures to the ultimate atomic thickness, beyond the state of the art of ultrasonics. Due to their strong and unique anisotropy, which affect most of the physical properties such as carrier diffusion and thermal conduction, opto-acoustic interactions can be truly confined to the atomic scale for the emission and detection of coherent phonons. This would yield ultimate out-of-plane spatial resolution and temporal bandwidth. The perfect mechanical coupling in heterojunctions of vdW layers already demonstrated and discussed in Section 4.5 further supports the feasibility of this approach.

These optimized schemes can make use of the nanofabrication state of the art and the mechanical and optoelectronic features previously discussed. One may think of the implementation of single layers as efficient transducers with atomic resolution and reduced influence on the overall acoustic behavior of a studied vdW heterostructure. Optimization of the opto-acoustic coupling may take advantage of the tunable, resonant, strong optical absorption of TMDs [16,17]. The large range of excitonic resonance energies available in the vdW materials family allows for selective addressing of individual layers in such heterostructure by tuning pump and probe beam wavelengths. In addition, the high efficiency of phonon generation based on the piezoelectric effect in III-V heterostructures [158,159] could be fruitfully put in relation with the strong piezoelectricity demonstrated in odd number of layers of MoS_2_ [106] and even stronger one calculated in some other vdW materials [264,265]. Interestingly, we note that hBN, which is the one vdW material usually used to encapsulate heterostructures, presents the lowest acoustic impedance in the reviewed list in Table 1. Consequently, it consistently yields a soft interface when in contact with another vdW layer and thus an anti-node for the acoustic oscillation. Yet, acoustic mismatch is relatively low with other reviewed vdW materials yielding significant transmission of acoustic energy through such heterostructure [136,137]. Hence, carefully chosen vdW materials and non-trivial architectures are required to reach confinement and shaping of acoustic oscillations in vdW heterostructures.

The development of optimized vdW transducers will have strong impact in the general field of time-domain Brillouin acoustics where depth profiling is performed in various systems, as developed in Section 3.2. As already demonstrated with a single layer of graphene [248], a vdW layer can be used in hybrid schemes to launch coherent acoustic phonons in another material. Following this discussion, engineered vdW heterostructures would allow emission, but also detection, with a better efficiency, spectral selectivity, at higher frequency, and with higher spatial resolution as compared to the currently utilized transducers. Furthermore, vdW layers present the advantage of yielding contaminant-free interfaces and very good conformation to surfaces even at the nanoscale, without requiring deposition techniques that can be harmful to fragile samples such as biological tissues, since transfer is performed in ambient conditions with simple micro-manipulators. Hence, vdW materials have the potential to bring out a novel generation of enhanced ultrathin transducers.

Additionally, coherent acoustic phonons may be utilized to introduce high frequency modulation up to several THz in the large variety of current and future optoelectronic devices based on vdW heterostructures. Acoustic tuning of the electronic, optical, magnetic, or structural properties is already widely implemented to create ultrafast modulators. In the case of vdW heterostructures, a high modulation efficiency should be obtained for features that strongly depend on the interlayer distance. Examples include the already discussed optical bandgap [17,19] and interlayer charge and energy transfer efficiency and speed in heterojunctions [219,220,221]. We also bring focus on other recently demonstrated highly interesting features related to interlayer coupling, such as electronic sub-band splitting stemming from out-of-plane confinement [266,267], room-temperature stable and in situ tunable interlayer excitons [268,269,270,271], interplay between superconductive and topological insulating phases in QLs [272,273], and correlated electronic states emerging from magic-angle twisted bilayers [274,275,276,277]. Ultrafast coherent modulation of these systems will open large opportunities in both fundamental and applicative investigations.

Finally, the foreseen improvements on the quality factor previously discussed based on improved interfaces and optimized architectures can take advantage from coupling with light and carriers. The investigation of strongly coupled optical and mechanical modes corresponds to a rich approach to yield all-optical actuation of nanomechanical motion, towards quantum-limited sensitivity [83,278]. Recent schemes showed optomechanical coupling in III-V slabs which does not require optical cavity [279] and would thus be compatible with current vdW nanofabrication techniques. Most promising approaches involve a mechanical nanoresonator coupled to a single two-level system [280,281], which can be obtained in vdW systems from intra- or interlayer excitons localized on defect [282], strain [283], or electrostatic [270] sites.

To conclude, in this review, we have shown that current investigations of coherent phonons in van der Waals systems already combine pioneering experimental results and important potential developments. In the same way as for time-domain electronic and more recently thermal studies on vdW heterostructures, there are large avenues to explore from fundamental characterization of vdW physics to implementation of enhanced device architectures. VdW systems have the potential to emerge as an ultimate platform for coherent phonon acoustics that can provide insights into unexplored mechanical behaviors based on the vdW coupling, a novel generation of superior opto-acoustic transducers, and optoelectronic devices coupled with nanoresonators for ultrafast THz modulation or towards quantum regimes.

## Figures and Tables

**Table 1:** Structural and mechanical parameters defined in the main text for a subset of relevant van der Waals materials. Values in bold are the one extracted from the indicated reference, and other mechanical parameters are deduced using Equations (1), (3) and (10). Values for non-layered materials used as substrate or capping layer in literature are also presented.

Material	Layer Structure	µ (×10^−6^ kg/m^2^)	d (Å)	ρ (×10^3^ kg/m^3^)	v_B_ (km/s)	K (×10^19^ N/m^3^)	C_33_ (GPa)	Z (×10^6^ Pa.s/m)	Source
graphene	1-atom	0.76	3.4	2.3	4.0	11.0	**37**	9.2	Neutron scattering [63]
					**4.1**	11.6	39	9.4	X-ray [73]
					4.0	10.9	**37**	9.1	Ultrasound [108]
					**4.0**	10.8	36	9.0	T-D ^1^ EM ^2^ [124]
hBN	1-atom	0.73	3.3	2.2	**3.4**	7.7	26	7.5	X-ray [72]
BP	2-atom	1.4	5.3	2.7	**5.3**	14.4	76	14	Propagative CAP [125]
					4.2	**8.8**	46	11	RCP anti-node [126]
					4.8	**11.8**	63	13	Theory [127]
					5.0	**12.7**	67	13	Raman [128]
					**4.6**	10.6	56	12	Ultrasound [129]
MoS_2_	3-atom	3.1	6.2	5.1	**3.5**	10.1	62	18	RCP anti-node ^3^
	trigonal				3.2	8.5	**52**	16	Neutron scattering [119]
					3.2	**8.6**	53	16	Raman [54]
					**2.9**	6.9	43	15	T-D ^1^ EM ^2^ [130]
					**2.5**	5.1	32	13	T-D ^1^ X-ray [131]
MoSe_2_	3-atom	4.5	6.5	6.9	**2.8**	8.5	55	19	RCP anti-node [132]
	trigonal				2.9	**9.3**	60	20	Raman [44]
MoTe_2_	3-atom	5.3	6.9	7.7	2.6	**7.8**	54	20	Raman [52]
	trigonal								
WSe_2_	3-atom	6.0	6.5	9.3	**2.5**	9.0	58	23	RCP anti-node [133]
	trigonal				2.4	**8.6**	56	23	Raman [54]
PtSe_2_	3-atom octahedral	4.8	5.0	9.5	**1.7**	5.6	28	16	RCP node [134]
					**1.8**	6.1	31	17	RCP anti-node [134]
					1.6	**5.1**	26	16	Raman [44]
GaS	4-atom	0.76	7.7	3.9	3.1	4.9	**38**	12	Brillouin spectroscopy [99]
InSe	4-atom	4.6	8.3	5.5	**2.5**	4.1	34	14	RCP node & anti-node [135,136,137]
					**2.6**	4.3	36	14	Ultrasound [109]
Bi_2_Se_3_	5-atom	7.3	9.5	7.7	**2.0**	3.2	31	15	Propagative CAP [39]
Bi_2_Te_3_	5-atom	7.8	10.2	7.6	**2.6**	5.1	52	20	Propagative CAP [138]
					**2.5**	4.6	46	19	RCP node [139]
					2.5	4.7	**48**	19	Ultrasound [140]
					2.6	5.0	**51**	20	Neutron scattering [141]
Re_6_Se_8_Cl_2_	clustered	6.9	9.1	7.6	**2.7**	6.1	55	21	Propagative CAP [142]
					**1.2**				Propagative CAP (shear) [142]
Al				2.7	6.4		111	17	
Sapphire				4.0	11.0		484	44	
Quartz				2.6	5.6		81	14	
Fused silica				2.2	6.0		78	13	
(100)Si				2.2	8.4		155	18	

^1^ T-D stands for time-domain. ^2^ EM stands for electron microscopy. ^3^ Acoustic velocity reported here is evaluated using data from [143] and Equation (9), while Equation (8) was used in the original work (see Section 4.4).
